# Long-Term Artificial Sweetener Acesulfame Potassium Treatment Alters Neurometabolic Functions in C57BL/6J Mice

**DOI:** 10.1371/journal.pone.0070257

**Published:** 2013-08-07

**Authors:** Wei-na Cong, Rui Wang, Huan Cai, Caitlin M. Daimon, Morten Scheibye-Knudsen, Vilhelm A. Bohr, Rebecca Turkin, William H. Wood, Kevin G. Becker, Ruin Moaddel, Stuart Maudsley, Bronwen Martin

**Affiliations:** 1 Metabolism Unit, Laboratory of Clinical Investigation, National Institute on Aging, Baltimore, Maryland, United States of America; 2 Section on DNA repair, Laboratory of Molecular Gerontology, National Institute on Aging, Baltimore, Maryland, United States of America; 3 Gene Expression and Genomics Unit, Laboratory of Genetics, National Institute on Aging, Baltimore, Maryland, United States of America; 4 Receptor Pharmacology Unit, Laboratory of Neurosciences, National Institute on Aging, Baltimore, Maryland, United States of America; 5 Bioanalytical Chemistry and Drug Discovery Section, Laboratory of Clinical Investigation, National Institute on Aging, Baltimore, Maryland, United States of America; Duke University, United States of America

## Abstract

With the prevalence of obesity, artificial, non-nutritive sweeteners have been widely used as dietary supplements that provide sweet taste without excessive caloric load. In order to better understand the overall actions of artificial sweeteners, especially when they are chronically used, we investigated the peripheral and central nervous system effects of protracted exposure to a widely used artificial sweetener, acesulfame K (ACK). We found that extended ACK exposure (40 weeks) in normal C57BL/6J mice demonstrated a moderate and limited influence on metabolic homeostasis, including altering fasting insulin and leptin levels, pancreatic islet size and lipid levels, without affecting insulin sensitivity and bodyweight. Interestingly, impaired cognitive memory functions (evaluated by Morris Water Maze and Novel Objective Preference tests) were found in ACK-treated C57BL/6J mice, while no differences in motor function and anxiety levels were detected. The generation of an ACK-induced neurological phenotype was associated with metabolic dysregulation (glycolysis inhibition and functional ATP depletion) and neurosynaptic abnormalities (dysregulation of TrkB-mediated BDNF and Akt/Erk-mediated cell growth/survival pathway) in hippocampal neurons. Our data suggest that chronic use of ACK could affect cognitive functions, potentially via altering neuro-metabolic functions in male C57BL/6J mice.

## Introduction

The epidemic of obesity and diabetes is currently one of the most important healthcare issues for both developed and emerging nations [1]. To limit excessive calorie intake, the use of artificial sweeteners has accelerated in recent decades. Between 1999 and 2004, more than 6000 new artificial sweetener-containing products were launched in the US alone [2]. Non-nutritive artificial sweeteners are considered to possess an ability to satisfy sweet taste sensation, while not contributing to caloric intake. Currently, 5 of these non-nutritive artificial sweeteners, acesulfame potassium (ACK), sucralose, aspartame, saccharin and neotame, are approved by the FDA [3]. Among them, ACK, discovered in 1967 [4], is approximately 200 times sweeter than table sugar. ACK has been approved for use in a variety of food products including carbonated drinks, baking products, baby food and frozen food. All currently approved non-nutritive sweeteners are recognized as safe, according to a 2004 American Dietetic Association report [5].

Besides initiation of lingual sweet sensation, there is recent evidence demonstrating that some artificial sweeteners can elicit other physiological actions such as altering incretin (GLP-1, GLP-2) secretion [6], [7], and modulation of glucose transport [8]. Some studies have also shown potential ACK-related genotoxicity effects [9], [10] and other cellular actions. In MIN6 pancreatic cells and rat pancreatic islets, ACK has been shown to induce insulin-secretion [11], [12]. ACK ingestion in murine models can also control intestinal expression levels of the sodium/glucose co-transporter (Slc5a1) in a similar manner to normal sugar [13]. Therefore, with respect to mimicry of the physiological effects of sugar, ACK also possibly possesses other activities in addition to its stimulatory sweet tasting capacity.

The potential link between the dietary use of artificial sweeteners and the generation of generic metabolic syndrome has not been convincingly investigated and thus still remains highly controversial. Swithers et al. reported that male rats given an ACK or saccharin-sweetened yogurt diet gained more weight than rats fed with a glucose- sweetened yogurt diet [14], [15]. However, a causal relationship between artificial sweetener intake and adiposity remains inconclusive, since the rats consuming saccharin-sweetened yogurt diet gained more lean mass instead of fat mass [15]. Similarly, inconsistent findings regarding the links between artificial sweeteners and metabolic homeostasis were also obtained in different human studies. Some epidemiological data has begun to demonstrate that the extent of consumption of artificial sweeteners, mainly in diet sodas, is positively associated with weight gain and an increased incidence of metabolic syndrome [16], [17]. For example, Fowler et al. found that artificially sweetened beverage consumption could fuel the obesity epidemic [18]. In contrast, however, de Koning et al. not only demonstrated that artificially sweetened beverage consumption was not associated with coronary heart disease (CHD) risk or intermediate biomarkers [19] but also highlighted the need for cautious interpretation of studies reporting positive associations between diet drinks and cardiometabolic and cardiovascular outcomes. These authors also point out that in the Health Professionals Follow-Up Study, participants appear to be consuming artificially sweetened beverages as part of a weight-loss strategy or in response to the diagnosis of a chronic condition. Taken together, the metabolic influence of use of artificial sweeteners is still debatable and mainly results from the difficulties of teasing apart a causative relationship versus reverse causality based on retrospective epidemiological studies.

In mammals, the sweetness of both nutritive, *e.g.* sucrose, and non-nutritive sweeteners such as ACK, is detected by the sweet taste receptor. The sweet taste receptor is a functional heterodimer formed by two G-protein coupled receptors *i.e.* the taste receptor, type 1, member 2 (T1r2) and the taste receptor, type 1, member 3 (T1r3) [20]. The taste preference for ACK in rodents is mainly dependent upon T1r3 subunit expression. In T1r3 knock-out (T1r3 KO) mice, the taste preference for ACK is remarkably lower than in wild-type (WT) animals [21]. Recent evidence has demonstrated that the T1r3 subunit is not only expressed in peripheral tissues such as the tongue, gut and pancreas [12], [20], [22], [23], but also in central nervous system (CNS) tissues including the hypothalamus, cortex and hippocampus [24], [25]. The presence of CNS T1r3 may provide a possible mechanism by which ACK may affect the central nervous system, in addition to its somatic actions. To better understand the actions of ACK in a mammalian biology system, we investigated both peripheral energy metabolism effects and potential CNS effects in response to ACK ingestion in a control murine population. Our study demonstrated that long-term (40 weeks) ACK ingestion elicited a moderate and limited influence on energy metabolic homeostasis such as alterations in levels of insulin and leptin with unclear clinical implications; most interestingly, the chronic intake of ACK significantly resulted in an impaired learning ability, which was potentially associated with deterioration of ‘neurometabolic’ functions in the brain.

## Results

### Physiological Effects of Extended ACK Ingestion

**Figure 1:**
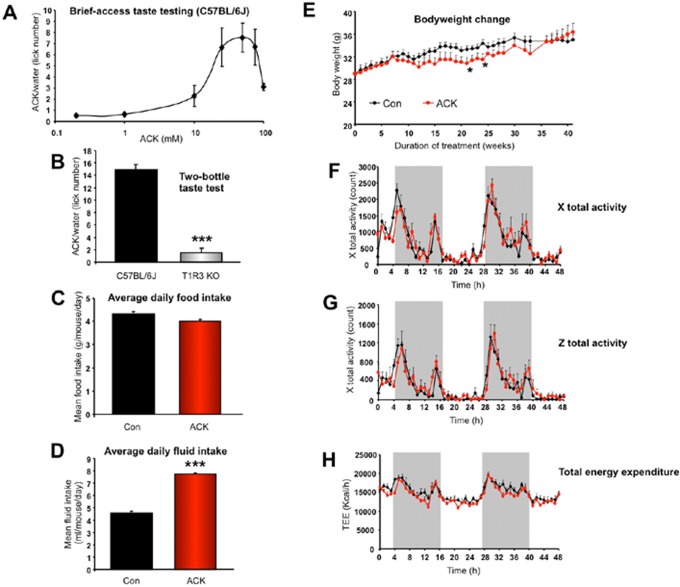
ACK taste responsivity and physiological alterations associated with ACK treatment. (A) Brief–access ACK taste response curve in WT(n = 6) mice. (B) 24 hour two-bottle taste preference test for ACK-supplemented water versus water-only control in WT(n = 8) and T1r3KO mice(n = 8). (C, D) Average daily food/fluid intake for water-treated (Con) or ACK-treated mice. (E) Bodyweight changes across study for water-treated or ACK-treated mice. (F) Ambulatory total X-axis activity in water-treated (black line) and ACK-treated (red line) mice. (G) Total Z-axis activity in water-treated (black line) and ACK-treated (red line) mice. (H) Total energy expenditure (TEE) in water-treated (black line) and ACK-treated (red line) mice. For panels F-H, activity was measured over a 48 hr period. In the chronic ACK treatment study, there are 6–8 mice for each group *i.e.* control (treated with water, n = 8) and ACK (treated with 12.5 mM ACK fluid, n = 6). A Student’s t-test was used for comparison between control and ACK treatment group and comparison between C57BL/6 mice and T1r3 KO mice. Data are means ± SEM. *p≤0.05, **p≤0.01, ***p≤0.001, n = 6–8/group.

As the tongue is one of the major peripheral organs responding to artificial sweeteners, we first investigated the taste responsivity of C57BL/6J (WT) mice to ACK-sweetened water using a brief access taste testing protocol. WT mice displayed an initial dose-dependent appetitive response to ACK concentrations up to 50 mM (Fig. 1A). Supramaximal ACK concentrations eventually induced an aversive response, an effect characteristic of many artificial sweeteners [26]. Based on this taste responsivity curve and existing literature reports [27], we chose 12.5 mM, which is close to our experimental EC_50_ value, as the dose for chronic ACK treatment. This dose of ACK for mice is within the expected exposure range for humans ingesting ACK. In order to exclude the possibility of aversive post-digestive effects, we employed a two-bottle taste-testing protocol to examine the long-term (24 h) preference of WT mice for 12.5 mM ACK-supplemented water. WT mice displayed a profound, 14-fold, preference for 12.5 mM ACK over water (Fig. 1B). Additionally, demonstrating a strong dependence of sweet taste ACK sensation upon T1r3 subunit activity [21], no significant preference for 12.5 mM ACK over water was observed using T1r3 KO mice in the two-bottle taste testing protocol (Fig. 1B).

With regards to the effects of ACK upon gross physiology, we found that ACK did not significantly change average daily food intake (Fig. 1C). ACK water supplementation however did induce a significantly increased fluid intake volume, compared to water (Fig. 1D). Despite the combination of no significant change in food intake, with an increased ACK-supplemented water intake, the mice demonstrated a relatively lower body mass over the majority of the study (weeks 10–35), compared to water-treated mice (Fig. 1E). Towards the end of the study, the mean weights between the water or ACK groups showed no significant difference (week 40) (Fig. 1E). When physical activity (x and z axis) was measured in both water and ACK-treated groups, we found no difference in activity levels across diurnal and nocturnal cycles (Fig. 1F, G). In line with this finding, we did not observe any ACK-mediated effects on total body energy expenditure (kCal/h) across diurnal/nocturnal cycles (Fig. 1H).

### Extended ACK Ingestion Alters Metabolic Hormone Levels

**Figure 2:**
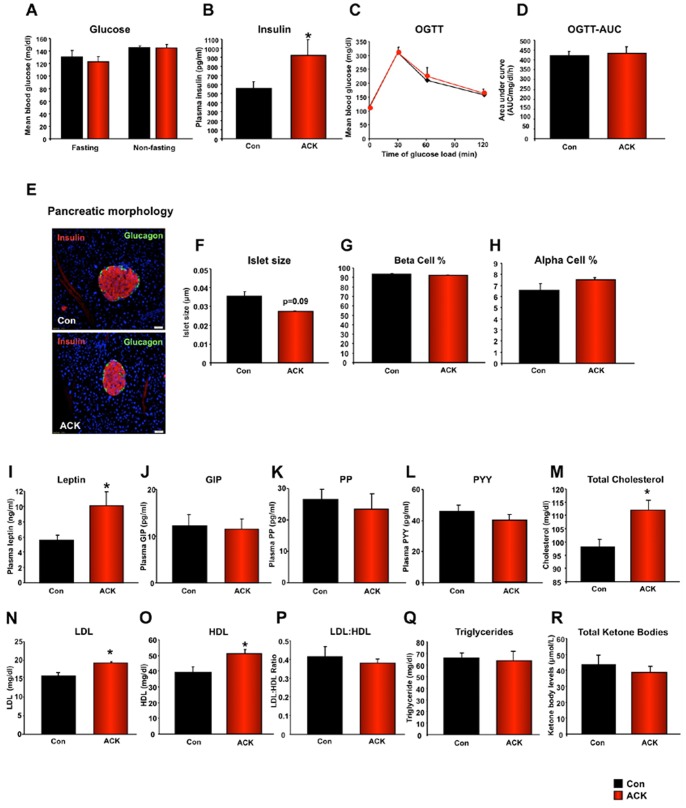
Extended ACK treatment alters metabolic factors. (A) ACK-mediated effects on fasting and non-fasting blood glucose in WT mice. (B) ACK-mediated effects on fasting insulin in WT mice. (C) ACK-mediated effects on the oral glucose tolerance test (OGTT) (water-treated control, black line: ACK-treated, red line). (D) ACK-mediated effects on OGTT AUC (Area Under Curve). ACK-mediated effects on pancreatic islet gross cellular morphology (E) and islet size (F). Insulin-secreting beta cells (red fluorescence) and Glucagon-secreting alpha cells (green fluorescence). Effect of ACK on pancreatic islet size (E), beta cell percentage (G) alpha cell percentage (H). Effects of ACK treatment on levels of leptin (I), gastrointestinal inhibitory peptide (GIP: J), pancreatic polypeptide (PP: K) and peptide YY (PYY: L). ACK effects on total plasma cholesterol (M). ACK effects on plasma lipoproteins: low-density lipoprotein (LDL: N); high-density lipoprotein (HDL: O); LDL:HDL plasma level ratio is indicated in panel (P). ACK effects on plasma triglycerides and ketone bodies (Q) and (R). A Student’s t-test was used for comparisons between control and ACK treatment group. Data are means ± SEM. *p≤0.05, n = 6–8/group.

With regards to the effects of extended ACK treatment upon euglycemic status, we found no significant difference between resting or fasting glucose levels for water or ACK-treated mice (Fig. 2A). However, ACK did cause a significant increase in the fasting circulating levels of insulin (Fig. 2B). ACK-treated mice shared similar glucose tolerance capacity with the water-treated mice in the Oral Glucose Tolerance Test (OGTT) (Fig. 2C, D). We next assessed any potential effect of ACK upon pancreatic islet morphology (Fig. 2E). We found that despite inducing no gross changes in islet ultrastructure, there was a moderate (not statistically-significant) reduction in total islet area of ACK-treated animals (Fig. 2F). ACK did not induce significant changes in beta (Fig. 2G) or alpha (Fig. 2H) cell islet area percentage. Global metabolic status however is not solely controlled by the pancreatic insulinotropic system, therefore we assessed the effects of ACK upon other circulating hormones that also affect energy metabolism. ACK treatment was found to significantly elevate circulating leptin levels (Fig. 2I). In contrast, ACK treatment caused no effect on plasma levels of gastric inhibitory polypeptide (GIP: Fig. 2J), pancreatic peptide (PP: Fig. 2K) or peptide YY (PYY: Fig. 2L). Elevated circulating adipokine levels, *e.g.* leptin, are often associated with changes in lipid metabolism [28]. We therefore investigated whether ACK affected circulating lipid profiles. ACK treatment induced a significant elevation in total cholesterol (Fig. 2M), LDL (Fig. 2N) and HDL levels (Fig. 2O). These complementary changes in lipoproteins therefore did not result in a significant ACK-mediated change in LDL:HDL ratio (Fig. 2P). ACK however failed to cause any global shift in triglyceride levels (Fig. 2Q) or ketone body levels (Fig. 2R).

### ACK Affects Neuronal Cellular Activity and Viability

**Figure 3:**
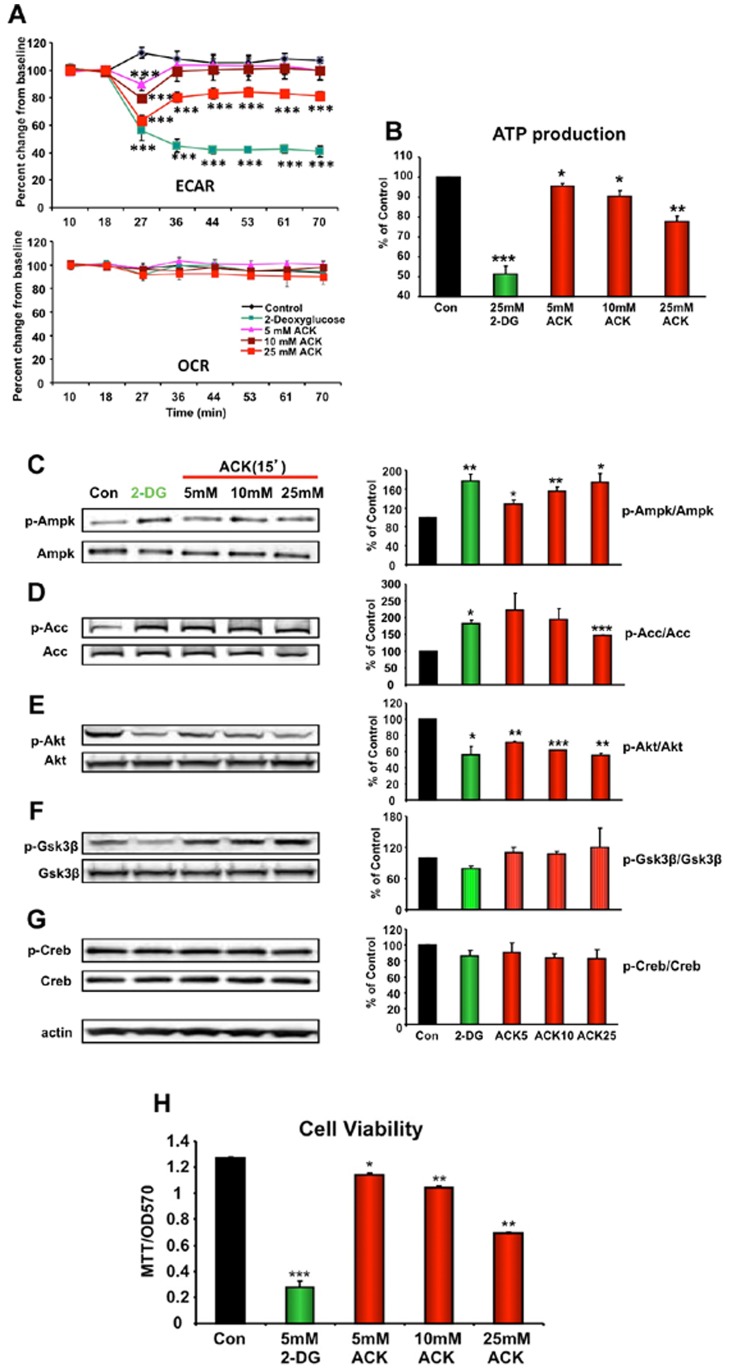
Alterations in cellular respirometry, ATP production, metabolic signaling factors and cell viability induced by ACK treatment of clonal neuronal cells. Dose-dependent effects of acute ACK exposure upon extracellular acidification rate (ECAR:A, upper panel) and oxygen consumption rate (OCR: A, lower panel) in neuronal cells. 2-DG, (25 mM), was used as a positive control metabolic toxin. (B) Acute ACK and 2-DG effects upon intracellular ATP production (C) were evaluated in SH-SY5Y cells after ACK acute treatment. Acute (15 min) ACK (5–25 mM) or 2-DG (25 mM) exposure-mediated changes in metabolic/survival protein expression in neuronal cells: phospho- (p-Ampk) and non-phosphorylated AMP-activated protein kinase (Ampk) (C); phospho- (p-Acc) and non-phosphorylated Acetyl-CoA carboxylase (Acc) (D); phospho- (p-Akt) and non-phosphorylated v-akt murine thymoma viral oncogene homolog 1 (Akt) (E); phospho- (p-Gsk3β) and non-phosphorylated glycogen synthase kinase 3β (Gsk3β) (F); phospho- (p-Creb) and non-phosphorylated cAMP response element-binding (Creb) (G). (H) ACK-mediated (24 h exposure) effects upon neuronal cell viability. Data from 3 independent experiments are expressed as means ± SEM. One-way ANOVA analysis was performed to evaluate changes in ECAR and OCR of each time point, alterations in intracellular ATP levels and cellular protein expression changes, where the treatment was one independent variable. p<0.05 was considered statistically significant throughout the study. Error bars represent the ±95% confidence interval. *p≤0.05, **p≤0.01, ***p≤0.001.

Considerable evidence has shown that even slight metabolic dysfunction can exert profound effects upon CNS tissues [29]–[31]. This is likely due to a combination of factors, *i.e.* the high energetic requirements of the CNS, combined with the high sensitivity of the post-mitotic neuronal tissues to metabolic stress [32], [33]. The existence of a brain T1r3 subunit may also provide a mechanism by which artificial sweeteners, such as ACK, can affect central nervous system activity. Thus, in the present study, the effects of ACK both acutely and chronically upon neuronal tissue were investigated, at both an *in vitro* and *in vivo* level. In the model neuronal cell line (SH-SY5Y) we detected the endogenous expression of T1r3 subunit (Fig. S1). As mitochondria form a vital functional metabolic locus in the CNS by generating the primary energy source, adenosine triphosphate (ATP), we further investigated the effects of ACK upon neuronal mitochondrial activity. We found that acute exposure of SH-SY5Y neuroblastoma cells to ACK caused a significant and dose-dependent reduction in mitochondrial extracellular acidification rate (ECAR), a proxy for functional mitochondrial integrity and glycolytic activity (Fig. 3A). With 5–10 mM ACK these inhibitory effects on ECAR were transient, but at higher concentrations (25 mM) ACK caused a protracted diminution of mitochondrial ECAR. The 25 mM ACK treatment effect upon ECAR was mimicked by cellular exposure to the metabolic toxin, 2-deoxyglucose (2-DG) (Fig. 3A, upper panel). In contrast, the neuroblastoma oxygen consumption rate (OCR) was not affected by any of the ACK concentrations (5–25 mM) (Fig. 3A, lower panel). Commensurate with the inhibitory actions of ACK and 2-DG upon ECAR, we found that neuronal ATP production was significantly attenuated in a dose-dependent manner by ACK and was nearly abolished by 2-DG (Fig. 3B). We next investigated the potential cellular signaling sequelae of these profound actions of ACK. Neuronal ACK exposure resulted in a significant dose-dependent increase in phosphorylation of the metabolic sensors 5′ adenosine monophosphate-activated protein kinase (Ampk) and Acetyl-CoA Carboxylase (Acc) (Fig. 3C, D). In contrast, neuronal ACK treatment significantly reduced the phosphorylation status of the neuroprotective kinase Akt (Fig. 3E). No significant changes were observed in the phosphorylation status of glycogen synthase kinase 3β (Gsk) or Creb (cAMP response element-binding) (Fig. 3F, G). For each of these indices, the molecular activity of 2-DG most closely mimicked that of the highest dose of ACK (25 mM). ACK-mediated increases in Ampk and Acc phosphorylation combined with decreases in Akt phosphorylation demonstrate the potential generation of cellular metabolic stress with a simultaneous inhibition of protective mechanisms, both of which may attenuate neuronal survival. When eventual cell viability was measured after 24 hr ACK exposure we found that both ACK (dose-dependent manner) and 2-DG significantly reduced neuronal cell viability (Fig. 3H). We found that acute ACK treatment inhibits glycolysis, decreases intracellular ATP production, and inhibits neuroprotective activity and cellular viability. It is interesting to note that many of these dysregulations are potentially associated with neurodegenerative diseases such as Alzheimer’s disease [32], [34]. We therefore next investigated whether ACK treatment generated a neurological phenotype.

### ACK Chronic Treatment in Mice Impairs Cognitive Function

**Figure 4:**
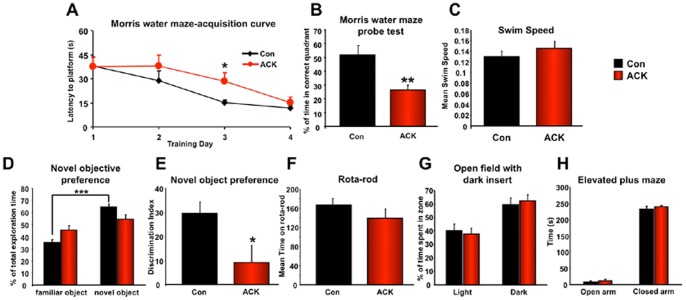
Extended exposure to ACK affects hippocampal-associated cognitive function in WT mice. (A) MWM acquisition was measured in mice exposed to the water treatment (Con) or ACK. (B) MWM probe test was also employed for water- and ACK-treated WT mice. (C) Effects of ACK on mean swim-speed was assessed across multiple MWM acquisition trials. (D) The percentage of total exploration time on familiar object and novel object in the NOP test were measured in water- or ACK-treated mice. (E) NOP discrimination index measured for water- or ACK-treated mouse behavior. (F) Rotarod performance of water- or ACK-treated wild-type mice. Open field with dark insert (G) and Elevated Plus maze performance (H) in water- or ACK-treated wild-type mice. Student’s t-test was used for comparison between control and ACK treatment group. Data are means ± SEM. *p≤0.05, **p≤0.01, ***p≤0.001, n = 6–8/group.

A primary tissue locus for age- and metabolism-related neuronal dysfunction is the hippocampus. Due to its high activity status and frequent strong calcium fluxes, this tissue often demonstrates excitoxicity as well as a large ATP demand. We investigated hippocampal function in the ACK-treated animals using Morris Water Maze (MWM) and Novel Object Preference (NOP) tests. ACK-treated mice, compared to water-treated mice, demonstrated a significantly slower MWM acquisition profile (Fig. 4A). The short-term memory retention (Probe Trial), measured 24 hrs after acquisition, in ACK-treated mice was also significantly lower than in water-treated mice (Fig. 4B). Despite these changes in cognitive performance, we found no significant ACK-mediated change in swim speed (Fig. 4C). ACK-treated mice also demonstrated a significantly lower NOP capacity (Fig. 4D) and NOP Discrimination Index (Fig. 4E) compared to water-treated mice**.** Interestingly, in a follow-up pilot study, with approximately 2-months of ACK treatment, ACK-treated T1r3 KO mice failed to demonstrate a declined cognitive function in the Morris Water Maze probe test compared to water-treated T1r3 KO mice. Meanwhile, male C57BL/6J mice under the same short-term ACK treatment consistently displayed a trend of memory impairment compared to their water-treated counterparts (Fig. S2). We found that the neurobehavioral effects of ACK were selective. There were no significant ACK-mediated effects upon motor coordination (rotarod task: Fig. 4F) or anxiety (open field with dark insert: Fig. 4G; elevated plus maze: Fig. 4H). Our results indicate that chronic ACK-treatment could elicit deleterious effects on the cognitive memory-related functions.

### ACK Treatment Causes Hippocampal Gene Transcript and Protein Alterations

**Figure 5:**
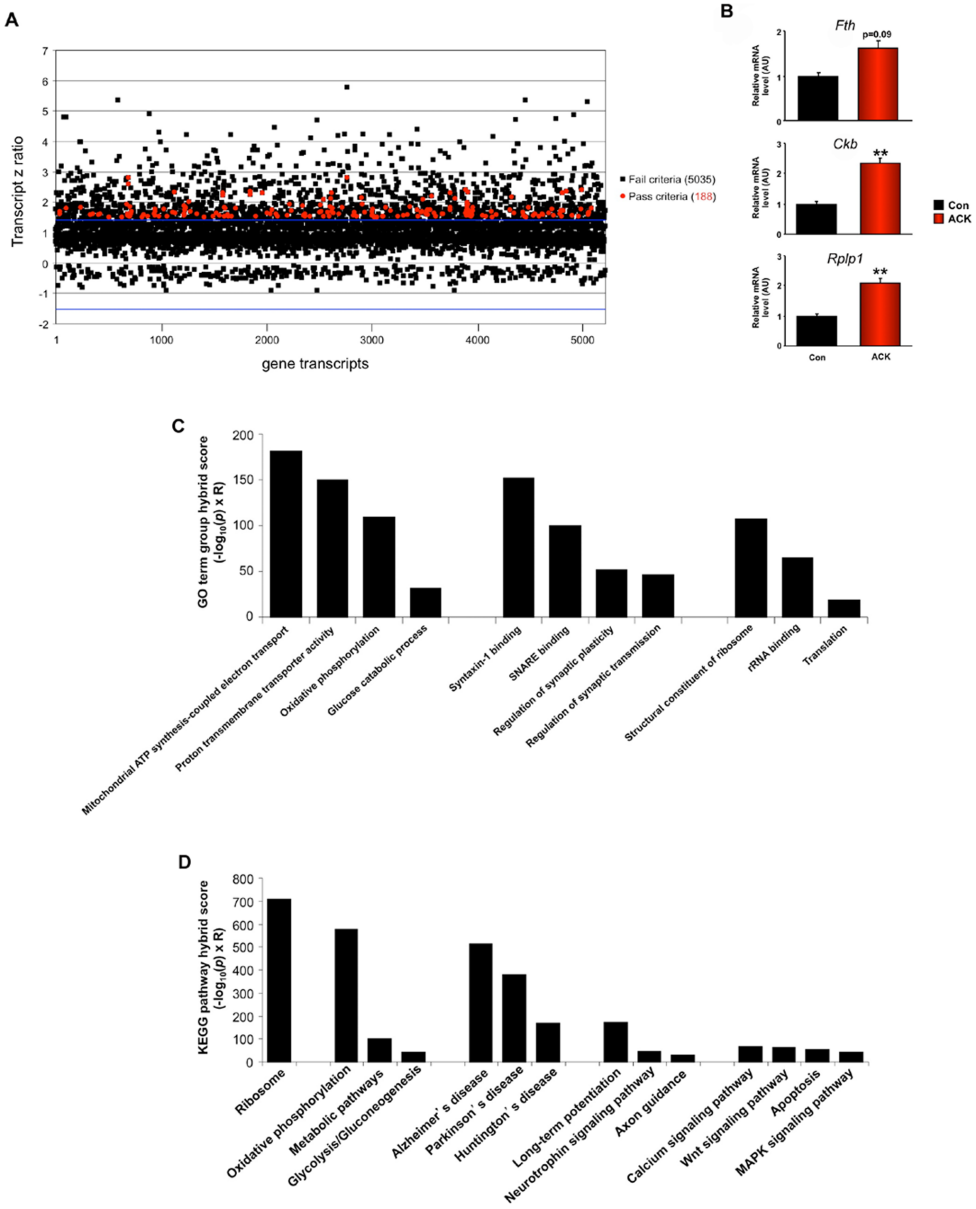
Hippocampal transcriptomic effects of extended ACK treatment. (A) Application of appropriate statistical criteria to differential (ACK versus water-treated) hippocampal transcriptomic data revealed the presence of 188 significantly ACK-regulated transcripts (red circles). Hippocampal transcripts identified that failed to meet statistical criteria are represented by black squares. Lateral blue lines indicate ±1.5 fold expression cut-off criteria. (B) Real-time PCR validation of selected array transcripts: Fth - ferritin heavy chain 1; Ckb - creatine kinase, brain; Rplp1 - ribosomal protein, large, P1. (C) Gene Ontology (GO) term enrichment of ACK significantly-regulated hippocampal transcripts. Histogram bars represent the hybrid score for each significantly-populated GO term group. (D) Kyoto Encyclopedia of Genes and Genomes (KEGG) pathway enrichment of ACK significantly-regulated hippocampal transcripts. Histogram bars represent the hybrid score for each significantly-populated KEGG signaling pathway. For significant GO term/KEGG pathway population, at least 5 distinct transcripts per GO/KEGG group and an enrichment probability of p≤0.01 were required.

In order to directly demonstrate ACK access to the brain in our study, we applied a HPLC-Mass spectrometric method (File S1) to quantitatively analyze brain ACK content. Three cortex samples of each group (control and ACK) from the same main chronic ingestive study were processed and analyzed. With the application of this HPLC-Mass spectrometric method, we were able to clearly demonstrate the presence of ACK in the cortex of ACK-treated mice (n = 3), the average value of which was around 10.54±3.83 µg/ml; whereas the values of controls were all below the detection limit (Fig. S3), since there were no co-elution peaks of ACK in all control samples. To investigate the potential molecular mechanisms associated with ACK-mediated memory impairment, we assessed the functional phenotypic transcriptomic signature of ACK in murine hippocampal tissue. We found 188 gene transcripts with statistically-significantly differential expression levels in ACK, compared to water-treated mice (Fig. 5A: Table S1). For validation of the transcriptomic array data we chose three random transcripts (ferritin heavy chain 1 (Fth1); creatine kinase, brain (Ckb); ribosomal protein, large, P1 (Rplp1)) on which we performed real-time PCR. Each PCR result (Fig. 5B) reliably recapitulated our array data (Table S2). To appreciate the potential functional effects of the interaction of these significantly-regulated transcripts, we performed Gene Ontology (GO) term enrichment analysis. We found that 3 generalized groups of enriched GO term clusters were significantly populated, *i.e.* metabolic activity, neurosynaptic and protein translational (Fig. 5C: Table S3). This molecular signature involving metabolic, neurosynaptic and translational emphasis was recapitulated when the ACK-sensitive transcriptome was clustered into KEGG (Kyoto Encyclopedia of Genes and Genomes) signaling pathways (Fig. 5D: Table S4). Indicating the negative cognitive effects of ACK treatment, we found the significant population of neurodegenerative disease pathways (*Alzheimer’s*, *Parkinson’s* and *Huntington’s disease*), neurodevelopmental effects (*long-term potentiation*, *neurotrophin signaling pathway*, *Wnt signaling pathway* and *axon guidance*) and signaling paradigms associated with excitoxicity (*calcium signaling pathway*, *apoptosis* and *MAPK signaling*). Using real-time PCR, we also evaluated hippocampal transcript changes of T1r1, T1r2 and T1r3. Compared to water-treated control animals, T1r1 mRNA expression was not significantly altered in the brain of ACK mice although there is a trend of ACK-induced up-regulation. A significant reduction in T1r2 mRNA levels was found in the hippocampus of ACK mice compared to controls (p<0.01, ACK vs. Control. Fig. S4). Intriguingly, the T1r3 mRNA expression was not profoundly changed by the ACK treatment.

**Figure 6:**
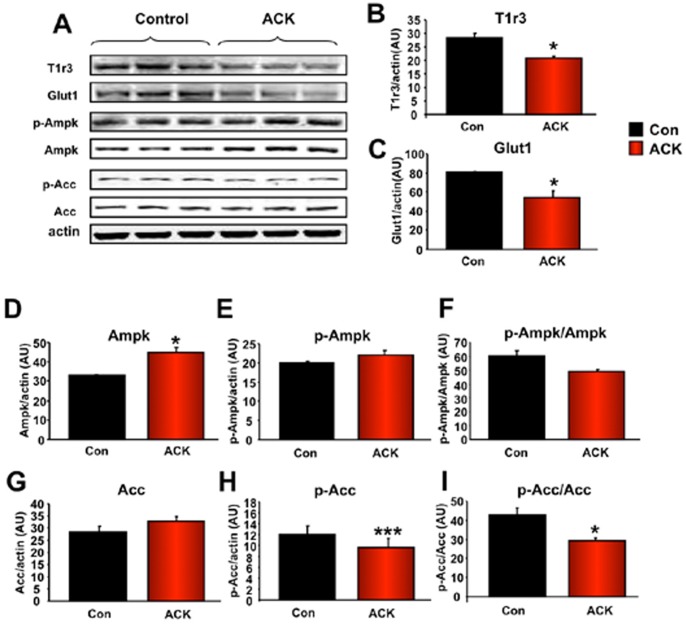
ACK-mediated alterations in hippocampal metabolism-associated proteins. (A) ACK-mediated alteration in expression levels of hippocampal proteins associated with energy metabolism were measured using western blot. (B) ACK-induced reduction of hippocampal sweet-taste receptor T1r3subunit (C) ACK-induced reduction of hippocampal glucose transporter 1 (Glut1/Slc2a1). ACK-mediated effects upon expression of Ampk (D), phosphorylated Ampk (p-Ampk: E) were measured to generate a p-Ampk/Ampk ratio (F). ACK-mediated effects upon the expression of Acc (G), phosphorylated Acc (p-Acc: H) were measured to generate a p-Acc/Acc ratio (I). Student’s t-test was used for comparison between control and ACK treatment group. Data are means ± SEM. *p≤0.05, **p≤0.01, ***p≤0.001, n = 3/group.

As the ACK hippocampal transcriptomic signature indicated that the pathophysiological effects of ACK upon memory formation are strongly associated with energy metabolism, neurodevelopmental and synaptic activity, we next investigated, at the protein level, specific factors that could mediate this specific molecular signature. Interestingly we found that two important components of the glucose sensing and catabolic/glucose uptake machinery were significantly down-regulated in response to ACK treatment, *i.e*. the glucose transporter Glut1 (Slc2a1) and the sweet taste sensory receptor T1r3 subunit (Fig. 6A–C). We also found that extended ACK treatment induced a significant increase in non-phosphorylated Ampk and a moderate increase in the phosphorylated form of Ampk (p-Ampk: Fig. 6A, E), which however resulted in a trend of a decrease in the p-Ampk/Ampk ratio (Fig. 6F). ACK treatment caused a moderate increase in the expression of Acc (Fig. 6A, G). Interestingly the Acc phosphorylation status (p-Acc) was significantly reduced by ACK treatment (Fig. 6A, H) which resulted in the creation of a significantly reduced p-Acc/Acc ratio (Fig. 6I).

**Figure 7:**
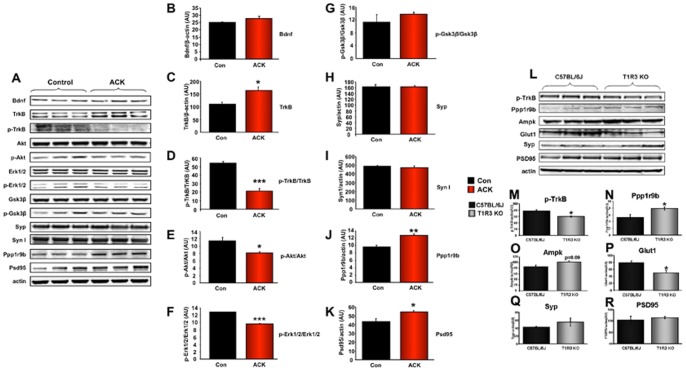
ACK-mediated alterations in hippocampal neurotrophic and synaptic proteins and neurometabolic alterations in T1r3 KO mice. (A) ACK-mediated alteration in expression levels of hippocampal proteins associated with neurotrophic activity and synaptic structure were measured using western blot. (B) ACK-mediated effects upon brain-derived neurotrophic factor (Bdnf) expression. (C) ACK-induced elevation of TrkB (Bdnf cognate receptor) expression. (D) ACK-induced reduction of activated TrkB receptor expression. TrkB receptor activation is indicated by its increase in tyrosine phosphorylation status. (E) ACK-induced reduction of phosphorylated Akt (p-Akt)/Akt expression. (F) ACK-induced reduction of phosphorylated Erk1/2 (p-Erk1/2)/Erk1/2 expression. (G) ACK-mediated effects upon phosphorylated Gsk3β (p-Gsk3β)/Gsk3β expression. ACK-mediated effects upon hippocampal synaptophysin (Syp: H), Synapsin I (Syn I: I), spinophilin (Ppp1r9b: J) and post-synaptic density protein 95 kDa (Psd95: K) expression. (L) Alterations in expression levels of hippocampal neuro-metabolic proteins were measured using western blot in T1r3 KO mice including the expression of phosphorylated TrkB (M), spinophilin (Ppp1r9b: N), Ampk (O), glucose transporter 1 (Glut1/Slc2a1, P), synaptophysin (Syp: Q), post-synaptic density protein 95kDa (Psd95: R), Student’s t-test was used for comparison between control and ACK treatment group and comparison between C57BL/6 mice and T1r3 KO mice. Data are means ± SEM. *p≤0.05, **p≤0.01, ***p≤0.001, n = 3/group.

As we observed a functional hippocampal memory-forming deficit and a degenerative neurosynaptic signature in the transcriptomic data, we further assessed the expression of proteins involved in neuronal survival, signaling and development. The hippocampal expression of the neurotrophic ligand, brain-derived neurotrophic factor (Bdnf), was moderately increased by ACK exposure (Fig. 7A, B). ACK treatment was also able to significantly upregulate the expression of the cognate receptor for Bdnf, *i.e.* TrkB (Fig. 7A, C). However, the functional activity of TrkB, measured via its tyrosine phosphorylation status (p-TrkB), was significantly attenuated by ACK (Fig 7A, D). Therefore it appears that multiple reactive pro- and anti-neurotrophic mechanisms were engendered by ACK treatment. Complex emergent systems such as cognitive networks often demonstrate multiple adaptive and reactive responses to neurological insults, and therefore often seemingly paradoxical protein expression data can be observed [35]. Reduced TrkB activity is strongly associated with advanced age and neurodegenerative phenotypes [32], therefore we assessed the expression of neuroprotective and developmental signaling molecules in the hippocampus. In line with our observation of reduced neurotrophic support, we found that ACK treatment led to significant reductions in both Akt and extracellular signal-regulated kinase 1/2 (Erk1/2) expression (Fig. 7A, E, F). In addition we found a non-significant trend for ACK treatment to increase the phosphorylation status of Gsk3β (Fig. 7A, G). Taken together, our investigation of the ACK-modulated protein expression in the hippocampus demonstrates reductions in metabolic support, reduced energy levels, blunted neurotrophic function and reductions in neuroprotective kinases, all of which may underpin the ACK-induced impaired neurometabolic status. As we observed significant changes in neurotrophic and neuroprotective proteins in the hippocampi of ACK-treated mice, we next assessed the expression of several hippocampal neurosynaptic marker proteins. ACK treatment did not appear to significantly affect the expression of synaptophysin (Syp: Fig. 7A, H) or synapsin I (Fig. 7A, I). In contrast we found that ACK treatment resulted in a significant increase of hippocampal expression of spinophilin (Ppp1r9b: Fig. 7A, J) and post-synaptic density protein 95kDa (Psd95: Fig. 7A, K). Our data indicate that ACK can simultaneously affect metabolic and neurotrophic functions in the hippocampus, which converge to result in cognitive deficits.

As we had previously demonstrated that our ACK-treatment paradigm reduced hippocampal T1r3 subunit protein expression, we investigated whether functional phenotypic similarities or differences may exist between ACK-treated WT mice and T1r3 KO mice. We found that several neurometabolic features, *i.e.* attenuated TrkB phosphorylation and Glut1 expression as well as increased spinophilin expression, were common to both ACK treatment and the T1r3 KO mice (Fig. 7). Indicative of the considerable differences in animal model status, *i.e*. congenital receptor knock-out versus drug-induced partial alterations of T1r3 subunit expression, we also uncovered differences in expression of proteins involved in neurometabolic function (Syp, Psd95 and Ampk) between ACK-treated and T1r3 KO mice (Fig. 7). Therefore clearly these two models may effect some similar neurometabolic actions, but they may be through partially-distinct molecular mechanisms.

## Discussion

We investigated both peripheral and central effects of protracted exposure to the widely-used, artificial, non-nutritive, sweet-tasting compound, ACK. While classically considered to be a sweet-receptor agonist, with less significant post-ingestive effects, we found that extended ACK exposure, at a dose frequently experienced by humans through dietary ingestion, in mice could moderately affect some circulating metabolic hormones including insulin and leptin (Fig. 2) without changing overall metabolic status such as insulin sensitivity and bodyweight. We also found that chronic ACK ingestion significantly impaired memory acquisition (Fig. 4), and caused neurometabolism-related genomic and proteomic alterations in the hippocampus (Fig. 5, 6). We also found that *in vitro*, ACK treatment could alter mitochondrial bioenergetics in neuronal-like cells *i.e.* SH-SY5Y cells (Fig. 3). The neurometabolic effects of ACK observed in the present study potentially converge to induce a phenotype that could resemble dysregulated neurological conditions.

To help curtail the current obesity epidemic, artificial sweeteners have been widely used as beneficial dietary supplements that provide sweet taste without adding extra caloric load. It has been estimated that in 2004, 12% of the US population used artificial sweeteners on a regular basis [36]. It is noteworthy that the five FDA-approved artificial sweeteners including acesulfame potassium (ACK), sucralose, aspartame, saccharin and neotame are quite different in compound structure and metabolic fates. It is highly possible that they or their metabolites can elicit diverse actions after they are administrated into the body, even if all of them can trigger lingual sweet taste sensation. For example, Aspartame is rapidly metabolized in the gastrointestinal tract by esterases and peptidases into three components: the two constituent amino acids, aspartic acid and phenylalanine, and methanol, in all species examined [37]. Different from ACK, Malaisse et al. found that Aspartame could not augment insulin release from rat islets incubated in the presence of 7.0 mM d-glucose [11], implying that it is not necessarily the case that all artificial sweeteners could pose equal health risks. In the present study, we chose to investigate, as our major focus, the long-term effects of ACK on both peripheral energy metabolism and CNS functions, as its primary target receptor, the sweet taste receptor, is expressed in energy-regulatory and CNS tissues.

Unlike aspartame, the sweetness of ACK can be detected by both humans and rodents [38], and studies have shown that taste sensation of ACK is mediated by the T1r3 subunit [20], which was confirmed by our observation of abolished taste responsivity to ACK in T1r3 KO mice (Fig. 1) [21]. We believe that the majority of the physiological effects observed in ACK-treated mice are largely idiosyncratic and substance-specific, *i.e.* other artificial sweeteners with diverse molecular structures may not reproduce the effects of ACK. Sucralose is another widely-used non-caloric artificial sweetener, which possesses a distinct chemical structure and metabolic fate from ACK. Following consumption, most sucralose (approximately 85%) is not absorbed, and is eliminated unchanged in feces [39]. Of the small amount of sucralose that is absorbed following consumption (approximately 15% of oral intake), most is also excreted unchanged [40]. As both sucralose and ACK can stimulate lingual sweet taste sensation, in one of our follow-up studies, we have also evaluated the physiological effects of chronic (40 weeks) sucralose consumption in male C57BL/6J animals. The results from that study demonstrated that protracted sucralose administration failed to significantly impair memory function in male C57BL/6J mice, compared to water-treated controls (Fig. S5A). Additionally, unlike ACK, chronic treatment of sucralose failed to induce any changes in fasting insulin (Fig. S5B) and leptin (Fig. S5C) levels of C57BL/6J mice. In contrast, in ACK-treated mice, elevated insulin and leptin levels were observed (Fig. 2).

The dose ranges of ACK we employed in this study are justifiable and potentially informative for human epidemiological research, based upon the recent consensus on animal-to-human dose transfer calculations [41]–[43]. Both the FDA and the Joint Expert Committee of Food Additions (JECFA) have set the Acceptable Daily Intake (ADI: *i.e.* the level that a person can safely consume everyday over their lifetime without risk) of ACK at up to 15 mg/kg/day [5]. However, due to the widespread inclusion of artificial sweeteners in many foodstuffs, the actual dosage levels experienced by people are extremely difficult to truly estimate. In our study, we used a 12.5 mM ACK solution, which was clearly appetitive (to a similar extent as sucrose, indicating an appropriate level of lingual taste stimulation) and consumed by the mice in an *ad libitum* manner. Similar dose levels have been employed by other research groups as well [27]. Based on the average daily fluid intake of the animals in our study [27], the approximate daily ACK intake was between 8.1 mg/mouse/day (231 mg/kg/day) and 12.5 mg/mouse/day (359 mg/kg/day). Using the well-accepted human equivalent dose (HED: [41]–[43]) calculation, the HED of ACK from our current study is in the range of 18.7 mg/kg/day −29.1 mg/kg/day, which is equal to only 1.2–1.9 fold of the human ADI. Thus, even with a different exposure paradigm, each animal in the study still encountered a similar level of ACK exposure each day as some humans do, when people follow the ADI guidance. Additionally, prior to the chronic treatment, a strong ACK-fluid preference was obtained in a two-bottle preference evaluation. Briefly, within a 24-hour testing period, both water and 12.5 mM ACK fluid were provided to all experimental animals, *i.e.* animals had free choice of both drink sources. All testing mice had *ad libitum* access to food, as well. The results showed that C57BL/6J mice demonstrated a strong preference for the consumption of ACK solutions over water, as reflected by the ratios of ACK to water in both total lick numbers (∼14 fold, Fig. 1B) and total drink volume (∼17 fold, data not shown). These results indicate that C57BL/6J mice would voluntarily and preferably drink more ACK, even when they were provided with water as an alternative choice. Based on a human-ADI-comparable dose and a strong preference of 12.5 mM ACK fluid, to a certain extent, the current long-term ACK fluid (only) exposure paradigm has its merits in mimicking a human lifestyle, in which people consistently use acceptable levels of ACK-sweetened beverages (including diet sodas and non-carbonated diet drinks) as their main daily fluid. Additionally, instead of following the FDA’s ADI recommendation, there is also a portion of the U.S. population that is likely to consume a level slightly in excess of the FDA ADI [37]. Therefore, the results from our study may be relevant to human circumstances of both normal and slightly excessive artificial sweetener users and the results generated from our study could have a high clinical relevance to human health. However, considering the average human would encounter ACK periodically throughout the day, a more clinically relevant administration paradigm such as a periodic treatment may enable us to better simulate the clinical conditions in our future studies.

We found that 40-week ACK-sweetened water treatment was capable of influencing both peripheral metabolic factors and brain functions, albeit to different extents. The peripheral metabolic impacts of ACK are relatively moderate and limited. Compared to general metabolic status, glucose levels and glucose tolerability, the levels of several circulating metabolic factors/hormones including insulin, leptin, total cholesterol and LDL were more significantly affected by long-term ACK treatment, albeit with unclear clinical significance. Intriguingly, a profound impairment in cognition due to ACK was also observed (Fig. 4). Further mechanistic investigation demonstrated that in neuronal models (*in vitro* and *in vivo*) expressing T1r3 subunit, ACK could directly affect neuro-metabolic activity via glycolysis inhibition, induce functional ATP depletion and attenuate cell growth/survival.

In terms of the influence on the bodyweight and energy expenditure/activity, again, we observed relatively moderate and limited effects in ACK-treated animals (Fig. 1). ACK-treated mice displayed slightly lower body mass than controls during the mid-phase of the study, however this difference was absent at the end of the study. We postulated that the ACK-mediated bodyweight reduction may, in-part, be due to their elevated leptin levels causing a small reduction in their daily food intake (Fig. 1, 2). As one of the metabolic hormones secreted by adipocytes, leptin is responsible for regulating food intake, body weight and energy homeostasis. We speculated that enhanced leptin levels in ACK mice might also be attributed to higher insulin levels instead of increased adiposity. By the end of the study, ACK-treated mice shared similar bodyweight with controls (Fig1D). Comparable levels of circulating triglycerides also implied similar amounts of adiposity between ACK-treated mice and controls (Fig 2). However, besides the total amount of adipose mass, leptin secretion is also affected by circulating insulin. Zeigerer et al. have shown that insulin regulates leptin secretion from 3T3-L1 adipocytes by a PI-3 kinase-independent mechanism [44]. Thus, we postulated that elevated fasting insulin levels might be one of potential causes of hyperleptinemia in the ACK-treated mice. In terms of the elevated fasting insulin levels observed in ACK mice, we believe this may be directly related to the insulin-secretion stimulatory action of ACK, which has been demonstrated in vitro by Malaisse et al. [11]. A glucose tolerance test was applied in the present study to evaluate insulin sensitivity. Comparable glucose tolerant abilities demonstrated unaltered insulin sensitivities in ACK-treated mice compared to water-treated controls. Thus, the finding of higher insulin levels (with intact insulin sensitivity) suggests that ACK may only affect insulin secretion without altering insulin sensitivity.

Although the brain constitutes only 2% of body mass, it accounts for 50% of total body glucose utilization [45]. In addition, the brain relies on glucose as its primary energy substrate in contrast to muscle and adipose tissue, which can also employ the catabolism of fatty acids or amino acids. As the CNS is more dependent upon glucose than other energetic organs, then its perturbation, via non-nutritive T1r3 subunit-interacting sweeteners, may be more profound. Underlining this potential effect of non-nutritive sweeteners upon CNS tissues, recent data has begun to demonstrate the expression of sweet-taste receptors in multiple regions of the CNS [25]. In a neuronal cell line we found that acute ACK exposure could inhibit glycolysis and reduce ATP production. This effect on neuronal energy status resulted in the activation of one of the master energy sensors - Ampk (Fig. 3). In these *in vitro* neuronal experiments ACK activity was mimicked by the glycolysis inhibitor 2-DG [46], [47]. Compromising the energy-generating capacity of neuronal cells is associated with subsequent cell survival; in line with this theory, we found that ACK exposure significantly inhibited the activity of the neuroprotective Akt kinase as well as obviously reduced physical cell viability (Fig. 3). 2-DG, which mimicked many of the cellular neuronal effects of ACK (Fig. 3), has indeed been shown to induce hypoglycemia in neuronal tissue via direct glycolysis inhibition [48]. The SH-SY5Y human neuroblastoma cell line is one of the most widely-used *in vitro* models employed to study the actions of metabolic stressors on neuronal function [49]. In our study, we revealed that acute ACK treatment profoundly affected glycolysis, which is reflected by the alterations of ECAR without altering the oxygen consumption rates (OCAR). To a certain extent, the changes of ECAR in ACK-treated cells showed similarities with 2-DG-treated cells. Multiple reports have demonstrated that 2-DG blocks glycolysis by inhibiting upstream glycolysis enzymes including hexokinase and phosphoglucose isomerase, the enzyme that mediates the conversion of glucose-6-phosphate to fructose-6-phosphate [50]. Thus, we speculated that the potential anaerobic mechanisms contributing to the effects of ACK on cellular energy production might involve the rapid but transient inhibition of rate-limiting enzymes of glycolysis such as hexokinase and phosphoglucose isomerase. It has been shown that SH-SY5Y cells could well recapitulate multiple neurometabolic features of neurons under multiple metabolic stress conditions [49], [51]–[52]. A recent study has demonstrated that in SH-SY5Y cells, both glycolysis and respiration pathways exist and both can contribute to intracellular ATP production [50]. Although we failed to observe significant alterations in oxygen consumption rate (OCR) induced by ACK, this does not necessarily mean that ACK had no affect on the respiratory pathway. In terms of changes in ECAR, ACK treatment elicited a rapid and transient effect. There were obvious dose-dependent recovery phases in the ECAR curves of ACK-treated cells, while in contrast 2-DG-treatment prevented any rapid recovery in ECAR. Additionally, the intracellular ATP levels we measured are most likely indicative of a final balance of both the ATP production and ATP consumption in the cell. Our data suggest that in the SH-SY5Y cells that possess glycolysis and respiration pathways, acute ACK treatment can result in intracellular ATP depletion/reduction or imbalance that is mediated by a rapid and transient glycolysis blockage action. These findings potentially have important translational implications, considering the fact that the brain contains bioenergetically heterogeneous cell populations such as neurons that rely strongly on respiration and glia that appear to primarily employ glycolysis-biased ATP-generating mechanisms [53]. Therefore distinct neurophysiological functions, attributed to either support glia or neurons, may underpin distinct CNS actions of chronic ACK use.

ACK treatment induced stress-related deterioration in a neuronal cell line led us to further investigate its effects upon CNS tissues with high energetic requirement, *i.e.* the hippocampus. We found that extended ACK exposure resulted in a dysfunction of leaning and memory when assessed using two different hippocampally-associated tasks, *i.e.* the MWM and NOP task. The ACK-mediated disruption of memory acquisition was not associated with any change in motor performance, physical activity or anxiety (Fig. 4). We investigated the selective hippocampal actions of ACK at the transcriptomic level to determine the subtle changes that may result in altered memory acquisition. An ACK treatment-induced hippocampal transcriptomic signature, generated by 188 significantly-regulated genes (Fig. 5), was uncovered. Using GO term and KEGG pathway bioinformatic analysis, we found that some of the most prominent functions associated with the ACK hippocampal molecular signature were associated with energy regulation, neurodegenerative disease and synaptic activity. Underpinning these functional predictions at the individual transcript level were multiple genes closely related to energy regulation (Ckb, Atp5B, Aldoc, Eno2, Ndufa13, Ndufb4: [54]–[59], cognitive-impairment associated neurodegeneration (Stxbp1, Itm2b, Cdk5r1, Ppp3ca, Hpca: [60]–[64] and neurotrophin/synaptic functionality (Elavl4, Ndrg4, Camk2b, Stmn1, Wasf1: [65]–[69]. Many of the functions of significantly-regulated genes in ACK-treated mice converge upon phenotypes of cognitive dysfunction associated with neuro-metabolic disruption. For example, upregulation of Atp5b is related to amyloid deposition in the brain of Tg2576 mice [70], whereas increased gene expression of ferritin heavy chain 1 (Fth1) indicated the protective response from neuronal cells when they are experiencing energy shortage [71].

Investigating the functional transcriptomic patterns in the hippocampus at the translated protein level, we found that ACK exposure resulted in expression alterations associated with both metabolic and functional neurosynaptic alterations. With respect to ACK-induced neurometabolic activity alterations we found that ACK caused a reduction in hippocampal T1r3 subunit and Glut1/Slc2a1 expression and a significant reduction in phosphorylated Acc levels (Fig. 6). The effects of ACK upon proteins associated with neurosynaptic function were more nuanced and possibly complicated by the interconnection between reactive and adaptive neuronal responses [32]. For example, we found that ACK reduced the functional signaling output for the Bdnf ligand, *i.e*. attenuated p-TrkB levels. However we also found that levels of Bdnf increased after ACK treatment. This seemingly paradoxical elevation of Bdnf may represent a reflexive dynamic response to the evident blunting of memory (Fig. 4) and neurotrophic signaling functions (Fig. 7) in ACK-treated mice. Perhaps linked to this anti-neurotrophic effect of ACK we also found significant diminutions of active pools of hippocampal Akt and Erk1/2 (Fig. 7). The murine hippocampus, experiencing the ACK exposure, appears to be attempting to mount a reactive response to the reduced Bdnf activity by also increasing the expression of the post-synaptic marker proteins spinophilin (Ppp1r9b) and post-synaptic density protein 95 kDa (Psd95) (Fig. 7). These complex results suggest that a dynamic interplay exists between hippocampal neuroprotective mechanisms and metabolic support.

The potential alterations in hippocampal neurometabolic status are likely to cause detrimental effects on the growth/survival of neuronal cells. Several pro-survival kinases such as Akt/Erk1/2 were inhibited by ACK exposure, which could also contribute to the neuronal dysfunction [72], [73]. Interestingly our observation of the ACK-mediated increased expression of the TrkB receptor itself, despite reductions in levels of phospho-TrkB, may also be indicative of ACK-induced neurometabolic stress as dynamic increases in TrkB are commonly associated with cerebral ischemia-induced metabolic deficits [74]. Thus, it is plausible that the hippocampus of ACK-treated animals may potentially have experienced continuous metabolic stress. In accordance with our observed cognitive impairment, the functional disruption of Bdnf signaling seemed to occur at the level of receptor functionality. Such neurotrophin signaling disruption has previously been implicated with age and disease-related cognitive decline paradigms [75].

A recent study conducted by Zhang et al. [76], has shown the sustained and detectable existence of ACK and its dynamic changes within 24 h in mouse amniotic fluid during pregnancy, or mother’s milk during lactation after a single oral infusion of ACK solution (20 mg). This study also demonstrated that even at 21 h after the oral infusion, there were still significant concentrations of ACK in both mouse milk and amniotic fluid samples, compared to water-treated controls. Additionally, from toxicity studies of ACK [77] it has been demonstrated that after oral administration, ACK is rapidly and completely absorbed and reaches a peak blood concentration within 30 min and then declines with a t_1/2_ of 4.8 hours in rats. At the time of maximum blood concentration, the highest levels of ACK were present in the gastrointestinal tract, bile, kidneys and bladder. These multiple lines of evidences indicate that ACK can be rapidly absorbed by the epithelium of the digestive tract and can enter the bloodstream, where it is then potentially transferred into other body fluids such as the amniotic fluid or breast milk. Considering the duration of treatment (40 weeks) and daily intake amount (12.58 mg), it is plausible that ACK might exist with significant amounts in blood, which could lead to the bioaccumulation of ACK in organs. In order to provide direct evidence showing ACK was also able to reach the brain in ACK-treated mice, we set up and applied a HPLC-Mass spectrometric method to quantitatively evaluate ACK bioaccumulation in brain tissue (Fig. S3). Our results successfully demonstrated a significant accumulation of ACK in ACK-treated mice brain samples compared to water-treated controls, the values of which were all below detection limit. It is noteworthy that all brain samples analyzed by HPLC-Mass spectrometry are from the same chronic ACK ingestion study, in which C57BL/6J mice received 12.5 mM ACK-sweetened water for 40 weeks. The observation of ACK bioaccumulation in the brain further supports the base of our hypothesis that, under the current treatment paradigm, ACK can cross the blood-brain barrier and potentially induce direct physiological alterations in the brain. In future studies, more extensive and quantitative analysis of ACK dynamic changes in both the circulation and brain will be conducted to give better insight into this issue.

In the present study, we also suggested that ACK-induced hippocampal T1r3 subunit suppression (Fig. 5) may be one of the potential mechanisms for the neurometabolic changes observed in ACK-treated mice. The results from our current study and a follow-up pilot study, to a certain extent, support this hypothesis. First, we found that the T1r3 KO mice failed to sense ACK in our standardized gustatory tests, which indicates that an intact and functional sweet taste receptor plays an important role in detecting lingual presence of ACK. Second, chronic ACK treatment attenuated the expression of the sweet taste receptor in the hippocampus, as reflected by the reduction of T1r3 protein expression and T1r2 transcript levels (Fig. S4). Most importantly, in a short-term ACK treatment pilot study, we found ACK-treated T1r3 KO mice did not show altered cognitive functions in the Morris Water Maze probe test compared to water-treated T1r3 KO mice. Meanwhile, male C57BL/6J mice under the same short-term ACK treatment already show a trend of memory impairment compared to their water-treated counterparts (Fig. S2). This hypothesis was also supported by the demonstration in T1r3 KO mice of similar neurometabolic protein alterations we observed in ACK-treated mice (Fig. 7L), *e.g.* decreased TrkB activity (p-TrkB: Fig. 7M), increased post-synaptic marker (Ppp1r9b: Fig. 7N), elevated total Ampk (Fig. 7O) and reduced glucose transporter (Glut1) (Fig. 7P). However, due to the significant mechanistic differences between the T1r3 KO and ACK-treated murine models, differences between ACK-treated and T1r3 KO mice were also noted, *e.g.* changes in the synaptic markers, Syp and Psd95 (Fig. 7Q, R). Despite the difference between genetic knockout and compound-induced partial receptor suppression, our results indicate that the potential interactions between neuronal functionality and T1r3 subunit-mediated metabolic modulation in CNS might play an important role in the neurological phenotype formation of ACK-treated mice. Based on these multiple lines of evidence, we therefore hypothesize that the sweet taste receptor, especially the T1r3 subunit plays an important role in detecting and responding to the exposure of ACK both in the peripheral and central nervous system (CNS). More investigations are clearly needed to expand upon this hypothesis in a greater depth. These issues will be more substantially addressed in our successive studies.

Interestingly, we found no significant decrease of T1r3 mRNA in the hippocampus of ACK-treated mice. In contrast, we found a pronounced reduction of T1r3 protein expression in hippocampus of ACK-treated mice. One potential reason for our findings may be linked to the effects of sustained ligand binding and activation upon receptor stability and down-regulation. For the majority of G protein-coupled receptors (GPCRs), ligand stimulatory events are terminated by various molecular mechanisms targeting the ‘active’ ligated GPCR to prevent excessive deleterious cell stimulation [78]–[81]. This process is generically termed agonist-induced desensitization. GPCRs are typically post-translationally modified (usually phosphorylation) to reduce G protein affinity while increasing the affinity for accessory proteins involved in further G protein-uncoupling (arrestins) and intracellular sequestration (*e.g*. adaptins and clathrin). After intracellular sequestration, GPCRs are then able to be either dephosphorylated and recycled to the plasma membrane to be receptive for additional stimuli (re-sensitization) or are targeted to degradative compartments within the cell (*e.g.* lysosomes or proteasomes) (down-regulation). Normal physiological levels of receptor stimulation typically do not induce the secondary down-regulatory pathway, however during times of excessive or repeated receptor stimulation; this pathway is specifically engaged to attempt to reduce the total cellular sensitivity to the potentially-deleterious levels of the stimulatory ligand. With chronic ACK exposure, it is possible therefore that while mRNA is being generated and the protein product is being produced and exported to the membrane at normal levels, the presence of repeated ACK exposure may induce excessive receptor down-regulation. Therefore with protracted binding and activation by long-term ACK exposure, it is plausible that protein expression of the sweet taste receptor T1r3 subunit was down-regulated, since the major binding site of ACK on the functional sweet taste heterodimer is located in the T1r3 subunit. Unlike endogenous ligands, the xenobiotic ligands (*e.g.* ACK compared to sucrose) for GPCRs are prone to demonstrate bias and potential “imbalances” in their signaling repertoire at the receptor and thus may ‘uncouple’ the sweet sensing response from the feedback of energy influx, which could also potentially contribute to the discrepant results from protein and transcript levels. In future studies, further investigation into the regulation of sweet taste receptor expression by artificial sweeteners will be addressed.

In summary, we demonstrated that long-term ingestion of ACK-sweetened water in male C57BL/6J mice led to neuronal metabolic (or ‘neurometabolic’) alterations and cognitive impairment. The generation of this phenotype is likely associated with the creation of neurometabolic and neurosynaptic instabilities in hippocampal neurons that regulate memory acquisition. Meanwhile, chronic ACK intake elicited relatively limited actions on the peripheral energy metabolism homeostasis, with unclear clinical implications.

## Materials and Methods

### Animals and ACK Treatment

Animal procedures were approved by the Animal Care and Use Committee of the NIA. In the brief-access taste testing protocol, 4-month old male C57BL/6J (n = 6) were evaluated. In the 24-h two-bottle taste preference test, 4-month old male C57BL/6J (WT) (n = 8) and T1r3 KO mice (n = 6) were provided with normal water and ACK fluid (12.5mM, Sigma). For the chronic (40-week) ACK treatment study, 4-month old male C57BL/6J mice were randomly divided into 2 groups (n = 6–8/group), then continuously provided with either 12.5 mM ACK fluid or normal water as their daily drink source. All animals had *ad libitum* access to the normal mouse chow. No animals were re-used in the different treatment paradigms. The original breeding pair of T1r3 KO mice was kindly provided by Dr. Charles Zuker (Columbia University). In exons 1–5 of those mice, encoding the N-terminal extracellular domain, a PGK-neomycin resistance cassette was inserted in (129X1/SvJ×129S1/Sv)F1- Kitl+-derived R1 embryonic stem (ES) cells. This strain was then backcrossed to C57BL/6 for two generations by the donating laboratory. In our study, this mixed genetic background was altered toward C57BL/6 through extensive and continuous backcrossing with C57BL/6J mice. The T1r3 KO mice in the current study were littermates from homozygous T1r3 KO breeding pairs. Their generation was F?+F5, meaning that after arrival in our animal facility, we interbred for 5 generations, although prior to that their history remains unknown.

During the chronic (40-week) ACK study, animal body weight, food and fluid intake were carefully measured and recorded by the same investigator on a weekly basis. All water bottles were closely monitored to avoid accidental leaks. Any food spillages of normal mouse chow were also carefully collected and measured by the same investigator. The total amount of chow spillages for each cage was deducted from the data of food intake. At the end of the study, animals were euthanized with isoflurane inhalation. Brains, pancreas and plasma were collected for analyses as described previously [75]. Three brain samples of each group (water- or ACK-treated) from the chronic ACK ingestion study were also analyzed for ACK content with a HPLC-Mass spectrometric method. Trunk blood was collected and centrifuged at 3,000 rpm, 4°C for 30 minutes to obtain plasma. In order to evaluate *in vivo* insulin sensitivity, an oral glucose tolerance test (OGTT) was performed. Briefly, all animals were fasted for 16 h, and were then given an oral glucose solution (2 g/kg bodyweight) by gavage. Blood glucose was measured by tail bleed at different time points (0 min, 30 min, 60 min and 120 min) after glucose loading.

### Taste Testing

A Davis MS-160 gustometer (DiLog Instruments, Tallahassee, FL) was used to assess taste responsivity and ACK dose determination. Mice were tested as previously described [82]. Briefly, mice were placed in the gustometer for 25 mins, and stimuli were presented in random order for 5 sec trials that were initiated by the mouse licking the stimulus spouts (ACK was presented as a concentration range of 0.2, 1.0, 10, 25, 50, 75, 100 mM or water). Additionally, a two-bottle taste preference test was also carried out as described previously [83]. Preference was characterized by calculating the ratio of ACK (12.5 mM) lick number to water link number over 24 h. The higher ratio indicates a higher preference for ACK. Briefly all mice were individually housed, provided with food *ad libitum* and presented with two sipper bottles for 24 hours. One bottle contained water and the other contained the tastant to be assessed *i.e.* 12.5 mM ACK. The positions of both bottles were switched every 12 hours. With a licking activity monitoring apparatus (Lafayette Instrument Company), lick numbers from each bottle of each cage were automatically recorded by the Activity Wheel Software (Lafayette Instrument Company) for 24 continuous hours. The total lick numbers were calculated based on the recoding data. The total consumed volume for each tastant was manually measured with a graduated cylinder. In order to minimize single-house-induced stress, all testing animals were housed individually for 24 hours before the test. On the test day, all experimental animals were allowed to acclimatize to the new environment for at least 1 h prior to the formal data collection.

### Metabolic Status Evaluations

A comprehensive animal metabolic monitoring system (CLAMS; Columbus Instruments, Columbus, OH) was used to evaluate total energy expenditure and activity. In order to minimize single-house-induced stress, all testing animals (n = 6–8/group) were housed individually for 24 hours before the test. On the test day, all experimental animals were also allowed to acclimatize to the new environment *i.e.* the metabolic cages for at least 2 hours prior to the formal data collection. Total energy expenditure (TEE) was calculated using [TEE = (3.815+1.232 *RQ) *VO_2_] and expressed as KCal/kg/h [84]. RQ is the ratio of VCO_2_ to VO_2_. VO_2_ and VCO_2_ represent the volume of O_2_ consumption and the volume of CO_2_ production, respectively. Activity was measured on x and z axes using infrared beams during a specified measurement period (48 h).

### Measurement of Metabolic Hormones and Lipids

Fasting and non-fasting glucose levels were measured using the EasyGluco blood glucose system (US Diagnostics, Inc.). Plasma metabolic hormones were measured using a rodent multiplex kit (Millipore, Billerica, MA), as described previously [85], [24]. Each sample was assayed in duplicate on a 96-well plate. Plasma lipid levels including triglycerides, total cholesterol, high density lipoprotein (HDL) and low density lipoprotein (LDL) were assayed according to the manufacturer’s instructions (Wako Chemicals USA, Inc., Richmond, VA).

### Insulin/Glucagon Immunohistochemistry and Pancreatic Islet Sizing

Pancreatic sections were immunostained for insulin and glucagon according to a previously described protocol [24]. Continuous pancreatic paraffin sections were incubated with the primary insulin antibody (guinea pig anti-swine insulin; 1∶500, Sigma-Aldrich) and glucagon antibody (1∶1000; mouse anti-glucagon, Sigma-Aldrich) overnight at 4°C and then incubated with secondary antibody (Alexa Fluor 488 goat anti–guinea pig, 1∶1000; or Alexa Fluor 568 goat anti–mouse, 1∶1,000; Invitrogen, Carlsbad, CA) for 1 h at room temperature. DAPI (1∶5000) was used to stain nuclear DNA. Sections were then imaged with an Olympus Fluoview IX70 microscope (Olympus America, Center Valley, PA). Quantification of immunohistochemistry images (islet sizing and percentage of alpha- and beta-cells) was performed in Matlab (Mathworks), as described previously [86].

### Behavioral Analyses

Morris water maze (MWM), Novel object preference (NOP), rotarod, elevated plus maze and open field with a dark insert tests were performed as described previously [75]. After 38-weeks of treatment, behavioral testing was carried out on all animals. All behavioral testing was completed at the same time each day. Morris water maze (MWM) testing was carried out to assess learning and memory ability. Briefly, animals received 4 days of acquisition training, consisting of four trials per day, with an inter-trial interval of approximately 10 min. Each trial lasted until the animal found the platform, or for a maximum of 1 min. Animals that failed to find the platform within 1 min were guided by the experimenter to the platform. For each trial, the mice were placed into the pool, facing the wall, with start locations varied pseudo-randomly. On the final day of testing (day 5), animals were given a probe trial in which the platform was removed and the amount of time spent in each quadrant was recorded over a 60 sec interval. This probe trial was performed 24 hours after the final day of training. The probe trial indicates whether the animal can remember where the escape platform was located. Swim speed of each animal during each trial was also recorded and analyzed. To confirm the MWM results, another test of learning and memory, the Novel object preference test (NOP), was carried out. Briefly, the animals were placed in opaque boxes (10 min) which contained two identical objects, and the time spent exploring each object was measured. Then, one of the objects was replaced with a new object, and again the time spent exploring each object was measured (10 min). Animals that do not exhibit memory impairment will spend more time exploring the novel object on the second phase of testing. Data was reported as time spent exploring each object and as the discrimination index (DI), *i.e.* the total time spent investigating the novel object/total time the animal spent investigating both objects. A lower discrimination index (DI) is indicative of an impaired ability to preferentially recognize the novel object. In addition to learning and memory, general behavioral indices were also assessed. The elevated-plus maze and open-field with dark insert tests were used to assess anxiety levels, as described previously [75], [87]. Briefly, mice were placed with their head in the dark area of the apparatus (ENV-510; MED Associates, St. Albans, VT) and their body in the light area of the apparatus, and allowed free exploration of both areas for 10 minutes. The time spent in each compartment was automatically recorded. For the elevated plus maze, the maze was elevated on a tripod 70 cm above the floor. Each mouse was placed in the neutral area in the center of the maze, and the time spent in open and closed arms was recorded for each animal during a single 5 min trial. Additionally, motor coordination was also tested using an accelerating rotarod system (Med. Associates, Georgia, VT). Briefly, the mice were trained to remain on the spinning rotarod apparatus during a 2 minute habituation trial (4 revolutions per minute (rpm)) on the day prior to the testing day. On test days, the mice were placed on the rotarod, which gradually accelerated from 4 to 40 rpm over a 5 minute time interval. The test was performed twice per day and the latency to fall was measured and averaged.

### Cell Culture

Human neuroblastoma SH-SY5Y cells were grown in MEM-F12(1∶1) medium containing 10% Fetal Bovine serum (Life Technologies, Gaithersburg, MD), 2 mM L-glutamine, 100 U/mL penicillin, and 100 µg/mL streptomycin (Life Technologies) in humidified, 37°C chambers with 5% CO_2_. When the cells reached 80–85% confluence, the media was replaced with FBS-free medium with different concentrations of ACK (5 mM, 10 mM and 25 mM) or 2-DG (25 mM). For western blot and ATP measurements, the duration of ACK treatment was 15 min.

### Cellular Respirometry and ATP Measurement

Respirometry of SH-SY5Y cells was performed using an XF24 Extracellular Flux Analyzer (Seahorse Bioscience, Billerica, MA) [88]. Briefly, the cells are seeded at a given density in Seahorse cell culture plates. The basal oxygen consumption (OCR) and extracellular acidification (ECAR) rates were measured to establish baseline rates. The cells were then metabolically perturbed by adding different concentrations of ACK (5 mM, 10 mM, 25 mM) or 2-DG (25 mM). Continuous measurements of both OCR and ECAR were performed after drug additions. The shifts in the cellular bioenergetic profile were characterized by percentage of change from baseline. All results were normalized by the final cell number. Intracellular ATP level was measured using luminescence ATP detection assay (ATPlite PerkinElmer Inc., Waltham, MA, USA) as per supplier's instruction. Briefly, SH-SY5Y cells (100,000 cells per 100 µL culture media/well) were grown in 96-well microplates and incubated with the different concentrations of ACK (5 mM, 10 mM and 25 mM) or 2-DG (25 mM) for 15 min. 50 µL of mammalian cell lysis solution which stabilizes the ATP was added. Then 50 µL of the substrate solution was added, and the microplate was shaken again for 5 min at 700 rpm. The plate was covered with an adhesive seal, dark-adapted for 10 min and luminescence was measured using a luminescence microplate reader. The ATP standard curve was made by plotting signal versus ATP concentrations. The signal for unknown samples was obtained by using a linear regression equation.

### RNA Extraction, Microarray, Bioinformatics Analysis and Real-time PCR

RNA isolation, subsequent cDNA generation, labeling and hybridization to Illumina Sentrix Mouse Ref-8 Expression BeadChips (Illumina, San Diego, CA) were carried out as previously described [89]. RT-PCR was used to validate the gene changes using gene-specific primer pairs (Table S4). Arrays were scanned using an Illumina BeadStation Genetic Analysis Systems scanner and the image data extracted using the Illumina BeadStudio v.3.0. KEGG (Kyoto Encyclopedia of Genes and Genomes: http://www.genome.jp/kegg/) pathway clustering was performed using WebGestalt algorithms (http://bioinfo.vanderbilt.edu/webgestalt), as described previously [34]. The degree of enrichment (R: expressed as a ratio) of KEGG pathway group was calculated as follows: R = O/E where O is the observed protein number in the KEGG pathway, E is the expected protein number in the KEGG pathway. P value calculated from the statistical test is given for the pathways with R>1 to indicate the significance of enrichment. Based on those parameters, a hybrid score is calculated with the formula of R*(−log_10_(P)). For Real-time PCR, firstly, a two step real-time reverse transcription (RT) was performed to reverse transcribe total RNA into cDNA. PCR reactions were carried out using gene-specific primer pairs (Table S4) and SYBR Green PCR master mix (Applied Biosystems, Foster City, CA, USA) using ABI prism 7000 sequence detection system (Applied Biosystems). The amplification conditions were 50°C (2 min), 95°C (10 min), and then 40 cycles at 95°C (15 s) and 60°C (1 min). The data were normalized to glyceraldehyde 3-phosphate dehydrogenase (Gapdh) mRNA. All real-time PCR analyses are represented as the mean ± SEM from at least three independent experiments, each performed in triplicate.

### Western Blot Analysis of SH-SY5Y Cells and Mouse Hippocampus

Cells were washed twice with PBS, and lysed in the following buffer (20 mM Tris, pH7.5, 150 mM NaCl, 2 mM EDTA, 2 mM EGTA, 1 mM sodium orthovanadate, 100 µM phenylmethylsulfonyl fluoride, 10 µg/mL leupeptin, 10 µg/mL aprotinin, 5 µg/mL pepstatin, 50 mM NaF, 1 nM okadaic acid and 0.5% NP-40). The lysates were sonicated for 10 seconds on ice, centrifuged at 16,000×g for 15 min, and supernatants were collected. Hippocampal tissues were processed using the Qproteome™ Cell Compartment Kit according to the manufacturer’s instructions (Qiagen, Valencia, CA). All protein extracts were quantified using BCA reagent (ThermoScientific, Rockford, IL) before resolution with SDS-PAGE and electrotransference to PVDF membranes (Perkin Elmer, Waltham, MA). Membranes were blocked for western blots as described previously [75] and primary antibody immune-reactive complexes were identified using alkaline phosphatase-conjugated secondary antisera (Sigma Aldrich, St. Louis, MO) with enzyme-linked chemifluorescence (GE Healthcare, Piscataway, NJ) and quantified with a Typhoon phosphorimager. Blots were probed with antibodies to phospho-Ser9-Gsk3β, Gsk3β, phospho-Ser473-Akt, Akt, phospho-Ser79-acetyl-coenzyme A carboxylase (p-Acc), acetyl-coenzyme A carboxylase (Acc), phospho-Thr172-Ampk, Ampk, phosphor-Ser133-Creb, Creb, phosphor-Tyr490-TrkB, TrkB, phospho-Erk1/2, Erk1/2, Syp, Syn1,Ppp1r9b and PSD95 (Cell Signaling Technology, Beverly, MA); T1r3 and Bdnf(Santa Cruz Biotechnologies, Dallas, TX). Glut1 antibody was purchased from EMD Millipore (Billerica, MA). Beta-actin (A5316) antibody was purchased from Sigma (St. Louis, MO).

### Statistical Analyses

Both the Student’s t-test and one-way ANOVA analyses were employed in this study. A Student’s t-test was used to evaluate all physiological and behavioral data, plasma parameters, real-time PCR and western blot results for comparison between ACK-treated mice and water-treated control mice as well as comparison between C57BL/6 mice and T1r3 KO mice. One-way ANOVA analysis (GraphPad Prism version 5) was also performed to evaluate changes in ECAR and OCR of each time point, alterations in the intracellular ATP levels and cellular protein expression changes, where the specific treatments were considered as one independent variable. p<0.05 was considered statistically significant throughout the study. Error bars represent the ±95% confidence interval. All data represent means ± S.E.M. (standard error of mean).

## Supporting Information

Figure S1 **Expression of T1r3 subunit in SH-SY5Y cells.** Representative western blot of endogenous T1r3 subunit expression in SH-SY5Y cells.
(TIF)

Figure S2 **Short-term exposure to ACK did not affect hippocampal-associated cognitive function in T1r3 KO mice.** In a follow-up pilot study, both C57BL/6J mice and T1r3 KO mice received approximately 2-months of ACK or water treatment. Morris Water Maze probe test was employed for evaluating cognitive functions in water- or ACK-treated WT mice or T1r3 KO mice. A Student’s t-test was used for comparison between control and ACK treatment group for each strain. Data are means ± SEM. *p≤0.05, **p≤0.01, ***p≤0.001, n = 6–8/group.
(TIF)

Figure S3 **Brain ACK measurement by HPLC-Mass spectrometric method.** A HPLC-Mass spectrometric method was applied to quantitatively analyze brain ACK content. (**A**) The representative HPLC-Mass chromatogram, in which red line stands for ACK-treated mouse brain sample and dark line indicates water-treated mouse brain sample**.** (**B**) The quantitative result of brain ACK content for both ACK-treated mice and control mice. A Student’s t-test was used for comparison between control and ACK treatment group. Data are means ± SEM. *p≤0.05, **p≤0.01, ***p≤0.001, n = 3/group.
(TIF)

Figure S4 **The effects of extended ACK treatment on hippocampal T1rs transcript levels.** Real-time PCR was used to evaluate hippocampal transcript changes of T1r1, T1r2 and T1r3. A Student’s t-test was used for comparison between control and ACK treatment group. Data are means ± SEM. *p≤0.05, **p≤0.01, ***p≤0.001, n = 3/group.
(TIF)

Figure S5 **The effects of long-term sucralose treatment on cognitive function, fasting insulin and leptin levels.** Male C57BL/6J mice were provided with normal drinking water (Con) or Sucralose-sweetened solution (1.25 mM) for 40 weeks. Standard Morris water maze probe test was applied to evaluate the alterations of cognitive function. The end-point fasting insulin and leptin levels were also analyzed by using a rodent multiplex kit (Millipore, Billerica, MA). (**A**) Chronic sucralose intake did not significantly impair cognition. By the end of study, (**B**) chronic sucralose intake did not significantly affect fasting insulin levels in male C57BL/6J mice. (**C**) Chronic sucralose intake induced a trend of decreased levels of leptin, compared to water-treated control mice. A Student’s t-test was used for comparison between control and Sucralose treatment group. Data are means ± SEM, n = 6–8/group.
(TIF)

Table S1 **Significantly-regulated hippocampal gene transcripts in ACK-treated compared to water-treated mice.** For each significantly regulated gene transcript, the Gene Symbol, textual definition and expression z ratio (ACK treated- versus water-treated) are represented.
(DOC)

Table S2 **Real-time PCR primers for transcript expression validation and T1rs evaluations.**
(DOC)

Table S3 **GO term enrichment analysis of ACK significantly-regulated hippocampal transcripts.** GO term (biological process+molecular function) annotation was performed using the significantly regulated murine hippocampal transcripts (Table S1) using the following pathway population criteria: number of genes/pathway ≥5, pathway enrichment probability ≤0.01. The column nomenclature is as follows: C - number of reference transcripts in the category; O - number of experimentally-observed transcripts per pathway; E – sample-size scaled expected number in the category; R – enrichment ratio; P – pathway enrichment probability (hypergeometric); H – hybrid pathway score ((−log_10_P) * R).
(DOC)

Table S4 **KEGG pathway analysis of ACK significantly-regulated hippocampal transcripts.** KEGG pathway annotation was performed using the significantly regulated murine hippocampal transcripts (Table S1) using the following pathway population criteria: number of genes/pathway ≥5, pathway enrichment probability ≤0.01. The column nomenclature is as follows: C - number of reference transcripts in the category; O - number of experimentally-observed transcripts per pathway; E – sample-size scaled expected number in the category; R – enrichment ratio; P – pathway enrichment probability (hypergeometric); H – hybrid pathway score ((−log_10_P) * R).
(DOC)

File S1 **Supplementary information of the ACK measurements in brain samples by a HPLC-Mass method.**
(DOC)
