# Supplementary figures and images for: Long-Term Artificial Sweetener Acesulfame Potassium Treatment Alters Neurometabolic Functions in C57BL/6J Mice
Source: PLoS One. 2013 Aug 7;8(8):e70257. doi: 10.1371/journal.pone.0070257 (PMC3737213; doi:10.1371/journal.pone.0070257)

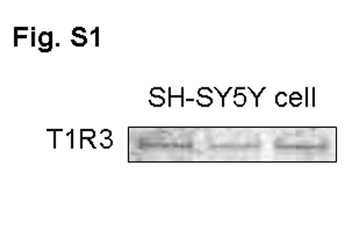

Supplement: Figure S1 — Expression of T1r3 subunit in SH-SY5Y cells. Representative western blot of endogenous T1r3 subunit expression in SH-SY5Y cells. (TIF) [file pone.0070257.s001.tif]

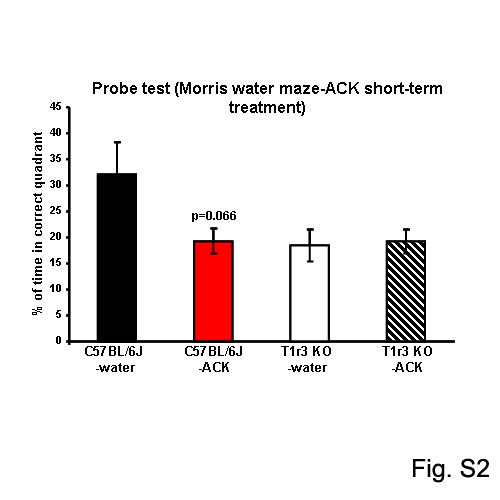

Supplement: Figure S2 — Short-term exposure to ACK did not affect hippocampal-associated cognitive function in T1r3 KO mice. In a follow-up pilot study, both C57BL/6J mice and T1r3 KO mice received approximately 2-months of ACK or water treatment. Morris Water Maze probe test was employed for evaluating cognitive functions in water- or ACK-treated WT mice or T1r3 KO mice. A Student’s t-test was used for comparison between control and ACK treatment group for each strain. Data are means ± SEM. *p≤0.05, **p≤0.01, ***p≤0.001, n = 6–8/group. (TIF) [file pone.0070257.s002.tif]

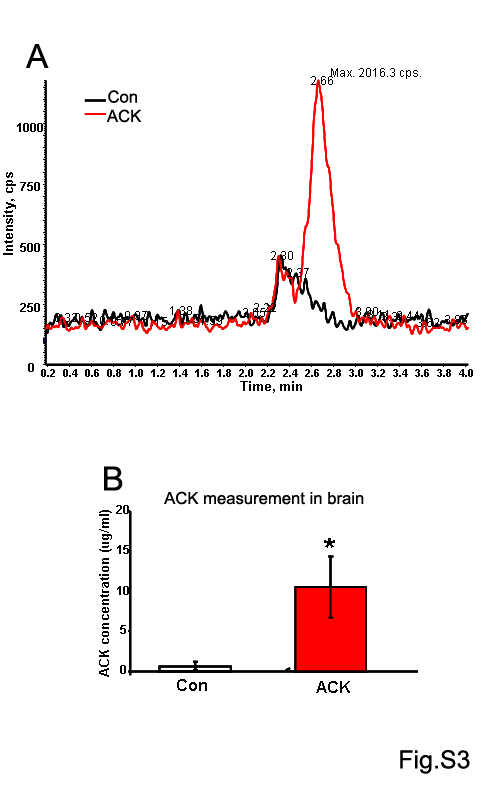

Supplement: Figure S3 — Brain ACK measurement by HPLC-Mass spectrometric method. A HPLC-Mass spectrometric method was applied to quantitatively analyze brain ACK content. (A) The representative HPLC-Mass chromatogram, in which red line stands for ACK-treated mouse brain sample and dark line indicates water-treated mouse brain sample. (B) The quantitative result of brain ACK content for both ACK-treated mice and control mice. A Student’s t-test was used for comparison between control and ACK treatment group. Data are means ± SEM. *p≤0.05, **p≤0.01, ***p≤0.001, n = 3/group. (TIF) [file pone.0070257.s003.tif]

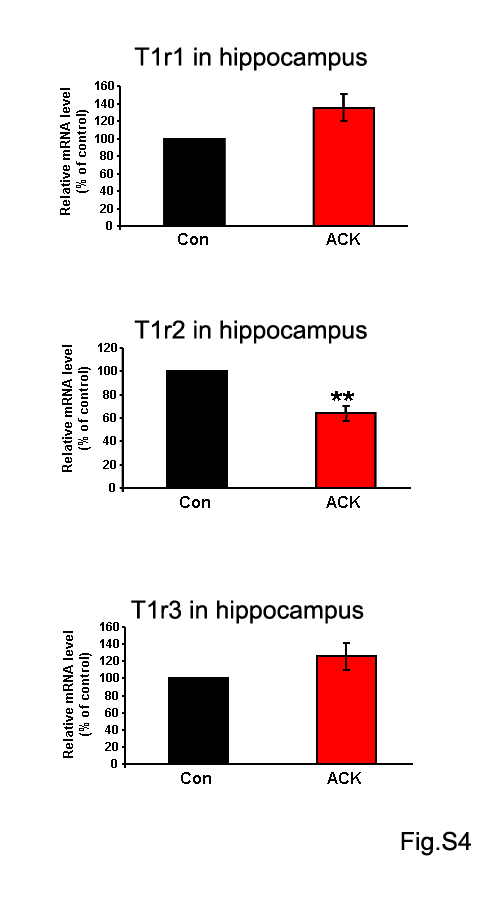

Supplement: Figure S4 — The effects of extended ACK treatment on hippocampal T1rs transcript levels. Real-time PCR was used to evaluate hippocampal transcript changes of T1r1, T1r2 and T1r3. A Student’s t-test was used for comparison between control and ACK treatment group. Data are means ± SEM. *p≤0.05, **p≤0.01, ***p≤0.001, n = 3/group. (TIF) [file pone.0070257.s004.tif]

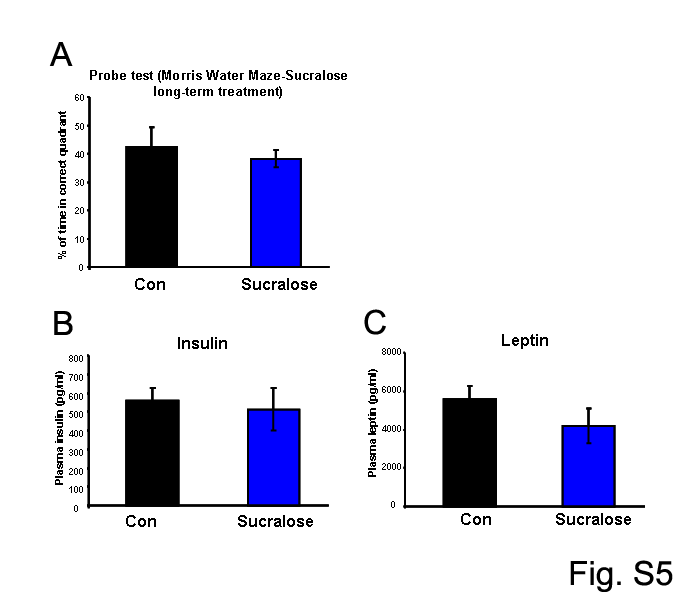

Supplement: Figure S5 — The effects of long-term sucralose treatment on cognitive function, fasting insulin and leptin levels. Male C57BL/6J mice were provided with normal drinking water (Con) or Sucralose-sweetened solution (1.25 mM) for 40 weeks. Standard Morris water maze probe test was applied to evaluate the alterations of cognitive function. The end-point fasting insulin and leptin levels were also analyzed by using a rodent multiplex kit (Millipore, Billerica, MA). (A) Chronic sucralose intake did not significantly impair cognition. By the end of study, (B) chronic sucralose intake did not significantly affect fasting insulin levels in male C57BL/6J mice. (C) Chronic sucralose intake induced a trend of decreased levels of leptin, compared to water-treated control mice. A Student’s t-test was used for comparison between control and Sucralose treatment group. Data are means ± SEM, n = 6–8/group. (TIF) [file pone.0070257.s005.tif]
