# Supplementary material for: Long-Term Artificial Sweetener Acesulfame Potassium Treatment Alters Neurometabolic Functions in C57BL/6J Mice
Source: PLoS One. 2013 Aug 7;8(8):e70257. doi: 10.1371/journal.pone.0070257 (PMC3737213; doi:10.1371/journal.pone.0070257)
Supplement: Table S1 — Significantly-regulated hippocampal gene transcripts in ACK-treated compared to water-treated mice. For each significantly regulated gene transcript, the Gene Symbol, textual definition and expression z ratio (ACK treated- versus water-treated) are represented. (DOC) [file pone.0070257.s006.doc]

**Table S1. Significantly-regulated hippocampal gene transcripts in ACK-treated compared to water-treated mice.** For each significantly regulated gene transcript, the Gene Symbol, textual definition and expression z ratio (ACK treated- versus water-treated) are represented.

| **Symbol** | **DEFINITION** | **z ratio** |
| --- | --- | --- |
| Ubb | ubiquitin B | 2.35 |
| Fth1 | ferritin heavy chain 1 | 2.35 |
| Ckb | creatine kinase, brain | 2.33 |
| Rplp1 | ribosomal protein, large, P1 | 2.32 |
| Eef1a1 | eukaryotic translation elongation factor 1 alpha 1 | 2.21 |
| Prnp | prion protein | 2.2 |
| Snurf | SNRPN upstream reading frame | 2.16 |
| Mt1 | metallothionein 1 | 2.14 |
| LOC218963 | ubiquitin pseudogene (LOC218963) on chromosome 14 | 2.11 |
| Rer1 | RER1 retention in endoplasmic reticulum 1 homolog (S. cerevisiae) | 2.11 |
| LOC100045981 | similar to synaptotagmin XI | 2.1 |
| Ppia | peptidylprolyl isomerase A | 2.1 |
| Mbp | myelin basic protein, transcript variant 7 | 2.08 |
| Atp5g3 | ATP synthase, H+ transporting, mitochondrial F0 complex, subunit c (subunit 9), isoform 3, nuclear gene encoding mitochondrial protein | 2.08 |
| Atp5b | ATP synthase, H+ transporting mitochondrial F1 complex, beta subunit, nuclear gene encoding mitochondrial protein | 2.06 |
| Wbp2 | WW domain binding protein 2 | 2.04 |
| Rpl23 | ribosomal protein L23 | 2.04 |
| Gm1821 | gene model 1821, (NCBI) (Gm1821) on chromosome 14 | 2.03 |
| Rasgrf1 | RAS protein-specific guanine nucleotide-releasing factor 1, transcript variant 1 | 2.03 |
| Eef1a1 | eukaryotic translation elongation factor 1 alpha 1 | 2.03 |
| LOC666904 | similar to heat shock protein 8 | 2.03 |
| Tuba1b | tubulin, alpha 1B | 2.03 |
| Cst3 | cystatin C | 2 |
| Rpl38 | ribosomal protein L38, transcript variant 1 | 1.99 |
| Rpl9 | ribosomal protein L9 | 1.94 |
| Atp1b1 | ATPase, Na+/K+ transporting, beta 1 polypeptide | 1.94 |
| Hsp90aa1 | heat shock protein 90, alpha (cytosolic), class A member 1 | 1.92 |
| Mtch1 | mitochondrial carrier homolog 1 (C. elegans), nuclear gene encoding mitochondrial protein | 1.92 |
| LOC100042427 | similar to Glyceraldehyde-3-phosphate dehydrogenase, transcript variant 4 | 1.89 |
| Rpl41 | ribosomal protein L41 | 1.88 |
| Cox6a1 | cytochrome c oxidase, subunit VI a, polypeptide 1, nuclear gene encoding mitochondrial protein | 1.87 |
| Itm2b | integral membrane protein 2B | 1.87 |
| LOC100048483 | similar to cytochrome c oxidase subunit VIII | 1.86 |
| Atp6v1e1 | VATPase, H+ transporting, lysosomal V1 subunit E1 | 1.84 |
| Pgls | 6-phosphogluconolactonase | 1.84 |
| Map1lc3b | microtubule-associated protein 1 light chain 3 beta | 1.83 |
| Syp | synaptophysin | 1.82 |
| Cplx1 | complexin 1 | 1.82 |
| 4833420G17Rik | RIKEN cDNA 4833420G17 gene | 1.82 |
| Nsf | N-ethylmaleimide sensitive fusion protein | 1.82 |
| Rasgrp1 | RAS guanyl releasing protein 1 | 1.81 |
| EG622339 | predicted gene, EG622339 | 1.81 |
| Rtn1 | reticulon 1, transcript variant 1 | 1.81 |
| Zcchc18 | zinc finger, CCHC domain containing 18, transcript variant 3 | 1.8 |
| 1810064F22Rik | RIKEN cDNA 1810064F22 gene | 1.8 |
| LOC675813 | similar to ribosomal protein S20 | 1.8 |
| Erdr1 | erythroid differentiation regulator 1 | 1.8 |
| EG668668 | predicted gene, EG668668 | 1.79 |
| Cpne6 | copine VI | 1.79 |
| Rpl37 | ribosomal protein L37 | 1.79 |
| Tpi1 | triosephosphate isomerase 1 | 1.78 |
| Cd81 | CD 81 antigen | 1.78 |
| Ndufa13 | NADH dehydrogenase (ubiquinone) 1 alpha subcomplex, 13 | 1.77 |
| Wasf1 | WASP family 1 | 1.77 |
| Camk2n1 | calcium/calmodulin-dependent protein kinase II inhibitor 1 | 1.76 |
| Cox7b | cytochrome c oxidase subunit VIIb, nuclear gene encoding mitochondrial protein | 1.75 |
| Snca | synuclein, alpha, transcript variant 2 | 1.75 |
| Rasgrf1 | RAS protein-specific guanine nucleotide-releasing factor 1 | 1.75 |
| Grina | glutamate receptor, ionotropic, N-methyl D-aspartate-associated protein 1 (glutamate binding) | 1.74 |
| Plp1 | proteolipid protein (myelin) 1 | 1.74 |
| Ppp3r1 | protein phosphatase 3, regulatory subunit B, alpha isoform (calcineurin B, type I) | 1.74 |
| Prkacb | protein kinase, cAMP dependent, catalytic, beta | 1.74 |
| Ttc3 | tetratricopeptide repeat domain 3 | 1.73 |
| Rps11 | ribosomal protein S11 | 1.73 |
| Htf9c | HpaII tiny fragments locus 9c, transcript variant 1 | 1.73 |
| Rps14 | ribosomal protein S14 | 1.73 |
| Ttc3 | tetratricopeptide repeat domain 3 | 1.72 |
| Rpl3 | ribosomal protein L3 | 1.72 |
| Tubb5 | tubulin, beta 5 | 1.72 |
| Ociad1 | OCIA domain containing 1 | 1.72 |
| Ppp3ca | protein phosphatase 3, catalytic subunit, alpha isoform | 1.72 |
| Rtn1 | reticulon 1, transcript variant 2 | 1.71 |
| Tuba1a | tubulin, alpha 1A | 1.71 |
| Ctnnd2 | catenin (cadherin associated protein), delta 2 | 1.7 |
| Sh3gl2 | SH3-domain GRB2-like 2 | 1.7 |
| LOC100043391 | PREDICTED: similar to QM protein | 1.7 |
| Ndg2 | Nur77 downstream gene 2 | 1.69 |
| LOC667250 | histone H3-like | 1.69 |
| Camta2 | calmodulin binding transcription activator 2 | 1.69 |
| 1110020P15Rik | RIKEN cDNA 1110020P15 gene | 1.69 |
| Atpif1 | ATPase inhibitory factor 1, nuclear gene encoding mitochondrial protein | 1.69 |
| Ppap2b | phosphatidic acid phosphatase type 2B | 1.68 |
| Rps2 | ribosomal protein S2 | 1.68 |
| Chst1 | carbohydrate (keratan sulfate Gal-6) sulfotransferase 1 | 1.67 |
| LOC100047615 | similar to 40S ribosomal protein S17 | 1.67 |
| Mlf2 | myeloid leukemia factor 2 | 1.67 |
| LOC100040592 | similar to Hmgcs1 protein, transcript variant 1 | 1.66 |
| Ywhaz | tyrosine 3-monooxygenase/tryptophan 5-monooxygenase activation protein, zeta polypeptide | 1.66 |
| Stk25 | serine/threonine kinase 25 (yeast) | 1.66 |
| Mxi1 | Max interacting protein 1, transcript variant 3 | 1.66 |
| Pom121 | nuclear pore membrane protein 121 | 1.66 |
| Psap | prosaposin | 1.65 |
| Ywhah | tyrosine 3-monooxygenase/tryptophan 5-monooxygenase activation protein, eta polypeptide | 1.65 |
| Tmsb4x | thymosin, beta 4, X chromosome | 1.65 |
| Dpysl2 | dihydropyrimidinase-like 2 | 1.64 |
| Sort1 | sortilin 1 | 1.64 |
| Lphn1 | latrophilin 1 | 1.64 |
| Rpl23 | ribosomal protein L23 | 1.63 |
| Got2 | glutamate oxaloacetate transaminase 2, mitochondrial, nuclear gene encoding mitochondrial protein | 1.63 |
| Cldn11 | claudin 11 | 1.63 |
| Ppp2ca | protein phosphatase 2 (formerly 2A), catalytic subunit, alpha isoform | 1.62 |
| Gprasp1 | G protein-coupled receptor associated sorting protein 1, transcript variant 3 | 1.62 |
| Aatk | apoptosis-associated tyrosine kinase | 1.62 |
| Actb | actin, beta, cytoplasmic | 1.62 |
| Rab2a | RAB2A, member RAS oncogene family | 1.62 |
| Cox4i1 | cytochrome c oxidase subunit IV isoform 1 | 1.61 |
| Calm3 | calmodulin 3 | 1.61 |
| Tmem10 | transmembrane protein 10 | 1.61 |
| Rab11b | RAB11B, member RAS oncogene family | 1.6 |
| Atp6v1g2 | ATPase, H+ transporting, lysosomal V1 subunit G2 | 1.6 |
| Kcna2 | potassium voltage-gated channel, shaker-related subfamily, member 2 | 1.6 |
| Rab11a | RAB11a, member RAS oncogene family | 1.6 |
| H1f0 | H1 histone family, member 0 | 1.6 |
| Tspan7 | tetraspanin 7 | 1.6 |
| Lypla2 | lysophospholipase 2 | 1.6 |
| D15Ertd682e | DNA segment, Chr 15, ERATO Doi 682, expressed | 1.6 |
| Terf2 | telomeric repeat binding factor 2 | 1.6 |
| 1110020P15Rik | RIKEN cDNA 1110020P15 gene | 1.6 |
| Atp2a2 | ATPase, Ca++ transporting, cardiac muscle, slow twitch 2 | 1.6 |
| Rreb1 | ras responsive element binding protein 1, transcript variant 1 | 1.59 |
| Acot7 | acyl-CoA thioesterase 7 | 1.59 |
| Per1 | period homolog 1 (Drosophila) | 1.59 |
| Slc1a3 | solute carrier family 1 (glial high affinity glutamate transporter), member 3 | 1.59 |
| A030009H04Rik | RIKEN cDNA A030009H04 gene | 1.59 |
| A2bp1 | ataxin 2 binding protein 1 | 1.58 |
| Mir16 | membrane interacting protein of RGS16 | 1.58 |
| Bex2 | brain expressed X-linked 2 | 1.58 |
| Tmem110 | transmembrane protein 110 | 1.58 |
| Ypel3 | yippee-like 3 (Drosophila) | 1.58 |
| Ppp3cb | protein phosphatase 3, catalytic subunit, beta isoform | 1.58 |
| Eif4a2 | eukaryotic translation initiation factor 4A2 | 1.58 |
| Rps7 | ribosomal protein S7 | 1.57 |
| Rps12 | ribosomal protein S12 | 1.57 |
| Ndufa4 | NADH dehydrogenase (ubiquinone) 1 alpha subcomplex, 4 | 1.57 |
| Jak1 | Janus kinase 1 | 1.57 |
| Atp2a2 | ATPase, Ca++ transporting, cardiac muscle, slow twitch 2 | 1.56 |
| Dynll1 | dynein light chain LC8-type 1 | 1.56 |
| Rpl31 | ribosomal protein L31 | 1.56 |
| Cox5b | cytochrome c oxidase, subunit Vb | 1.56 |
| Ndrg4 | N-myc downstream regulated gene 4 | 1.56 |
| Hpca | hippocalcin | 1.56 |
| Camk2b | calcium/calmodulin-dependent protein kinase II, beta | 1.56 |
| Pdcd2 | programmed cell death 2 | 1.56 |
| Trappc4 | trafficking protein particle complex 4 | 1.56 |
| Stmn1 | stathmin 1 | 1.55 |
| Atp6ap2 | ATPase, H+ transporting, lysosomal accessory protein 2 | 1.55 |
| Tceal5 | transcription elongation factor A (SII)-like 5 | 1.55 |
| Uqcrh | ubiquinol-cytochrome c reductase hinge protein | 1.55 |
| Mif | macrophage migration inhibitory factor | 1.55 |
| Tcf25 | transcription factor 25 (basic helix-loop-helix), transcript variant 1 | 1.55 |
| Sec14l2 | SEC14-like 2 (S. cerevisiae) | 1.55 |
| Hba-a1 | hemoglobin alpha, adult chain 1 | 1.54 |
| Stxbp1 | syntaxin binding protein 1 | 1.54 |
| Vps28 | vacuolar protein sorting 28 (yeast) | 1.54 |
| EG625054 | predicted gene, EG625054 on chromosome 17 | 1.54 |
| Morf4l2 | mortality factor 4 like 2 | 1.54 |
| Dnajc6 | DnaJ (Hsp40) homolog, subfamily C, member 6 | 1.54 |
| Ttc3 | tetratricopeptide repeat domain 3 | 1.54 |
| Thy1 | thymus cell antigen 1, theta | 1.54 |
| Nptn | neuroplastin | 1.54 |
| Gapdh | glyceraldehyde-3-phosphate dehydrogenase | 1.54 |
| Ndufb4 | NADH dehydrogenase (ubiquinone) 1 beta subcomplex 4, nuclear gene encoding mitochondrial protein | 1.53 |
| Npc2 | Niemann Pick type C2 | 1.53 |
| Klc1 | kinesin light chain 1, transcript variant a | 1.53 |
| LOC100046207 | similar to Lymphocyte antigen 6H precursor (Ly-6H) (LOC100046207) | 1.53 |
| Sepm | selenoprotein M | 1.53 |
| Atp5j2 | ATP synthase, H+ transporting, mitochondrial F0 complex, subunit f, isoform 2 | 1.53 |
| Ccdc117 | coiled-coil domain containing 117 | 1.53 |
| LOC100048105 | similar to Ubc protein, transcript variant 1 (LOC100048105) | 1.53 |
| Atp6v1b2 | ATPase, H+ transporting, lysosomal V1 subunit B2 | 1.53 |
| Kcnab2 | potassium voltage-gated channel, shaker-related subfamily, beta member 2 | 1.53 |
| Btbd10 | BTB (POZ) domain containing 10 | 1.52 |
| Psmb4 | proteasome (prosome, macropain) subunit, beta type 4 | 1.52 |
| Cdk5r1 | cyclin-dependent kinase 5, regulatory subunit (p35) 1 | 1.52 |
| Rps9 | ribosomal protein S9 | 1.52 |
| Pacsin1 | protein kinase C and casein kinase substrate in neurons 1 | 1.52 |
| Elavl4 | ELAV (embryonic lethal, abnormal vision, Drosophila)-like 4 (Hu antigen D), transcript variant 1 | 1.52 |
| Pmpcb | peptidase (mitochondrial processing) beta, nuclear gene encoding mitochondrial protein | 1.52 |
| Eno2 | enolase 2, gamma neuronal | 1.52 |
| Aldoc | aldolase 3, C isoform | 1.51 |
| Bcas1 | breast carcinoma amplified sequence 1 | 1.51 |
| Ndufb8 | NADH dehydrogenase (ubiquinone) 1 beta subcomplex 8 | 1.51 |
| Surf4 | surfeit gene 4 | 1.51 |
| Neurl | neuralized homolog (Drosophila) | 1.5 |
| Arl8a | ADP-ribosylation factor-like 8A | 1.5 |
| Ddx17 | DEAD (Asp-Glu-Ala-Asp) box polypeptide 17 (Ddx17), transcript variant 1 | 1.5 |
| Sharpin | SHANK-associated RH domain interacting protein | 1.5 |
