# Supplementary material for: Long-Term Artificial Sweetener Acesulfame Potassium Treatment Alters Neurometabolic Functions in C57BL/6J Mice
Source: PLoS One. 2013 Aug 7;8(8):e70257. doi: 10.1371/journal.pone.0070257 (PMC3737213; doi:10.1371/journal.pone.0070257)
Supplement: Table S2 — Real-time PCR primers for transcript expression validation and T1rs evaluations. (DOC) [file pone.0070257.s007.doc]

**Table S2. Real-time PCR primers for transcript expression validation and T1rs evaluations.**

| **Gene name** | **Accession number** | **Forward primer (5’-3’)** | **Reverse primer (5’-3’)** |
| --- | --- | --- | --- |
| ferritin heavy chain 1 (Fth1) | NM_010239.1 | CAAGTGCGCCAGAACTACCA | ACAGATAGACGTAGGAGGCATAC |
| creatine kinase, brain (Ckb) | NM_021273.3 | CCAACAGCCATAATACGCAGA | GCTGCTCAGATCAGGGAACT |
| ribosomal protein, large, P1 (Rplp1) | NM_018853.3 | CTCGCTTGCATCTACTCCGC | GGCATTGATCTTATCCTCCGTGA |
| taste receptor, type 1, member 1 (T1r1) | NM_031867 | CCAGCAGTCCCAATCAGC | CCAGCCTTGTGGAACAGA |
| taste receptor, type 1, member 2 (T1r2) | NM_031873 | AGGCTTTCTTCACCGTTTG | CCGTTGCGGTAGTTAGGG |
| taste receptor, type 1, member 3 (T1r3) | NM_031872 | GCAGATGGGTGCTATCCT | GCTTTCTTGGCATTCCTT |
