# Supplementary material for: Long-Term Artificial Sweetener Acesulfame Potassium Treatment Alters Neurometabolic Functions in C57BL/6J Mice
Source: PLoS One. 2013 Aug 7;8(8):e70257. doi: 10.1371/journal.pone.0070257 (PMC3737213; doi:10.1371/journal.pone.0070257)
Supplement: Table S3 — GO term enrichment analysis of ACK significantly-regulated hippocampal transcripts. GO term (biological process+molecular function) annotation was performed using the significantly regulated murine hippocampal transcripts (Table S1) using the following pathway population criteria: number of genes/pathway ≥5, pathway enrichment probability ≤0.01. The column nomenclature is as follows: C - number of reference transcripts in the category; O - number of experimentally-observed transcripts per pathway; E – sample-size scaled expected number in the category; R – enrichment ratio; P – pathway enrichment probability (hypergeometric); H – hybrid pathway score ((−log10P) * R). (DOC) [file pone.0070257.s008.doc]

**Table S3. GO term enrichment analysis of ACK significantly-regulated hippocampal transcripts.** GO term (biological process + molecular function) annotation was performed using the significantly regulated murine hippocampal transcripts (Table S1) using the following pathway population criteria: number of genes/pathway ≥5, pathway enrichment probability ≤ 0.01. The column nomenclature is as follows: C - number of reference transcripts in the category; O - number of experimentally-observed transcripts per pathway; E – sample-size scaled expected number in the category; R – enrichment ratio; P – pathway enrichment probability (hypergeometric); H – hybrid pathway score ((-log_10_P) * R).

| **GO term description** | **GO ID** | **C** | **O** | **E** | **R** | **P** | **H** |
| --- | --- | --- | --- | --- | --- | --- | --- |
| mitochondrial ATP synthesis coupled electron transport | GO:0042775 | 10 | 5 | 0.09 | 45.42 | 0.0001 | 181.68 |
| hydrogen ion transmembrane transporter activity | GO:0015078 | 73 | 11 | 0.63 | 17.36 | 2.14E-09 | 150.504 |
| oxidative phosphorylation | GO:0006119 | 48 | 8 | 0.42 | 18.93 | 1.78E-06 | 108.8395 |
| glucose catabolic process | GO:0006007 | 52 | 5 | 0.46 | 10.92 | 0.0011 | 32.30799 |
| syntaxin-1 binding | GO:0017075 | 8 | 5 | 0.07 | 43.2 | 0.0003 | 152.1884 |
| SNARE binding | GO:0000149 | 17 | 5 | 0.15 | 27.1 | 0.0002 | 100.2421 |
| regulation of synaptic plasticity | GO:0048167 | 38 | 5 | 0.33 | 14.94 | 0.0003 | 52.63181 |
| regulation of synaptic transmission | GO:0050804 | 70 | 7 | 0.62 | 11.36 | 0.0001 | 45.44 |
| structural constituent of ribosome | GO:0003735 | 134 | 14 | 1.16 | 12.04 | 1.32E-09 | 106.9083 |
| rRNA binding | GO:0019843 | 24 | 5 | 0.21 | 19.2 | 0.0004 | 65.24045 |
| translation | GO:0006412 | 364 | 15 | 3.21 | 4.68 | 0.0001 | 18.72 |
