# Supplementary material for: Long-Term Artificial Sweetener Acesulfame Potassium Treatment Alters Neurometabolic Functions in C57BL/6J Mice
Source: PLoS One. 2013 Aug 7;8(8):e70257. doi: 10.1371/journal.pone.0070257 (PMC3737213; doi:10.1371/journal.pone.0070257)
Supplement: Table S4 — KEGG pathway analysis of ACK significantly-regulated hippocampal transcripts. KEGG pathway annotation was performed using the significantly regulated murine hippocampal transcripts (Table S1) using the following pathway population criteria: number of genes/pathway ≥5, pathway enrichment probability ≤0.01. The column nomenclature is as follows: C - number of reference transcripts in the category; O - number of experimentally-observed transcripts per pathway; E – sample-size scaled expected number in the category; R – enrichment ratio; P – pathway enrichment probability (hypergeometric); H – hybrid pathway score ((−log10P) * R). (DOC) [file pone.0070257.s009.doc]

**Table S4. KEGG pathway analysis of ACK significantly-regulated hippocampal transcripts.** KEGG pathway annotation was performed using the significantly regulated murine hippocampal transcripts (Table S1) using the following pathway population criteria: number of genes/pathway ≥5, pathway enrichment probability ≤ 0.01. The column nomenclature is as follows: C - number of reference transcripts in the category; O - number of experimentally-observed transcripts per pathway; E – sample-size scaled expected number in the category; R – enrichment ratio; P – pathway enrichment probability (hypergeometric); H – hybrid pathway score ((-log_10_P) * R).

| **KEGG Pathway** | **C** | **O** | **E** | **R** | **P** | **H** |
| --- | --- | --- | --- | --- | --- | --- |
| Ribosome | 125 | 14 | 0.34 | 41.75 | 1.06E-17 | 708.6935 |
| Oxidative phosphorylation | 164 | 15 | 0.44 | 34.1 | 1.06E-17 | 578.8371 |
| Metabolic pathways | 1229 | 25 | 3.3 | 7.58 | 4.54E-14 | 101.1395 |
| Glycolysis / Gluconeogenesis | 110 | 5 | 0.3 | 13.56 | 0.0006 | 43.68827 |
| Alzheimer's disease | 265 | 19 | 0.71 | 26.73 | 6.17E-20 | 513.4757 |
| Parkinson's disease | 174 | 13 | 0.47 | 27.85 | 2.63E-14 | 378.2042 |
| Huntington's disease | 231 | 11 | 0.62 | 17.75 | 3.56E-10 | 167.7118 |
| Long-term potentiation | 81 | 6 | 0.22 | 27.62 | 5.09E-07 | 173.8205 |
| Neurotrophin signaling pathway | 144 | 5 | 0.39 | 12.94 | 0.0002 | 47.86467 |
| Axon guidance | 139 | 5 | 0.37 | 10.73 | 0.0015 | 30.30054 |
| Calcium signaling pathway | 203 | 7 | 0.54 | 12.86 | 7.06E-06 | 66.24437 |
| Wnt signaling pathway | 160 | 6 | 0.43 | 13.98 | 2.28E-05 | 64.89607 |
| Apoptosis | 95 | 5 | 0.25 | 15.7 | 0.0003 | 55.3092 |
| MAPK signaling pathway | 281 | 7 | 0.75 | 9.29 | 4.94E-05 | 40.00528 |
