# Supplementary material for: Long-Term Artificial Sweetener Acesulfame Potassium Treatment Alters Neurometabolic Functions in C57BL/6J Mice
Source: PLoS One. 2013 Aug 7;8(8):e70257. doi: 10.1371/journal.pone.0070257 (PMC3737213; doi:10.1371/journal.pone.0070257)
Supplement: File S1 — Supplementary information of the ACK measurements in brain samples by a HPLC-Mass method. (DOC) [file pone.0070257.s010.doc]

**Supplementary information of the ACK measurements in brain samples by a HPLC-Mass method**

**1.1 Instrumentation**

The chromatographic experiments were carried out on a Shimadzu Prominence HPLC system (Shimadzu, Columbia, MD, USA). The samples were introduced to the analytical column using Shimadzu SIL-20A autosampler and maintained at 4˚C in the autosampler tray, and injections of 20 µl were made.

**1.2 Chromatographic conditions**

Acesulfame K (ACK) was eluted using an Eclipse XDB-C18 guard column (4.6 mm x 12.5 mm) and an Agilent Zorbax XDB-C8 3.5 µm analytical column (4.6 x 75 mm) purchased from Agilent (Santa Clara, CA). The internal standard used was sodium Cyclamate (200 µg/ml). The mobile phase consisted of water containing 0.1% trifluoroacetic acid as Component A and methanol containing 0.1% trifluoroacetic acid as component B. A linear gradient was run as follows: 0 min 40% B; 7.5 min 70% B; 10min 40% B and 15 min 40% B at a flow rate of 0.4 ml/min. The total run time was 15 min per sample.

**1.3 Mass spectrometry conditions**

MS/MS analysis was performed using a triple quadrupole mass spectrometer model API 5500 system from Applied Biosystems/MDS Sciex equipped with Turbo Ion Spray® (TIS) (Applied Biosystems, Foster City, CA, USA). The data was acquired and analyzed using Analyst version 1.4.2 (Applied Biosystems). Negative electrospray ionization data were acquired using multiple reaction monitoring (MRM). The TIS instrumental source settings for temperature, curtain gas, ion source gas 1 (nebulizer), ion source gas 2 (turbo ion spray), and ion spray voltage were 550˚C, 40 psi, 70 psi, 70 psi and -4000 V, respectively. The TIS compound parameter settings for declustering potential, entrance potential, collision energy and collision cell exit potential were -60 V, -10 V, -19V and -10 V, respectively for ACK and -120V, -10 V, -40 V and -10V, respectively for cyclamate. The standards were characterized using the following MRM ion transitions: ACK (161.2/82.0); cyclamate (179.5/181.8).

**2. Sample preparation**.

When brain samples were analyzed, the cortex was weighed and suspended in 230 µl of Dulbecco's Phosphate Buffered Saline (D-PBS) 1X, without calcium or magnesium and 20 µl of 10 mg/ml of cyclamate (IS) was added. The solution was homogenized on ice with a polytron homogenizer and centrifuged 16,100 rcf (relative centrifugal force) for 10 min. The supernatant was transferred to an autosampler vial for analysis.

**3. Validation**

**3.1 Stock solution**

A concentrated stock solution was prepared containing 20 mg/ml of ACK. The solution was prepared in ultra-pure water and stored in polypropylene tubes at -20˚C. Serial dilutions of the stock solution were used to prepare the samples for the calibration curves and quality control standards (QC).

**3.2 General procedures**

The standards used in the calibration studies as QC standards for the analysis of ACK ranged from250µg/ml to 3.91µg/ml. The quantification of ACK was accomplished using area ratios calculated using cyclamate as the internal standard and calculating per mg of tissue. Quality control standards were prepared by adding 20 µl of the corresponding spiking standard solution and 20 µl of internal standard to control brains suspended in 210 µl of D-PBS 1X without calcium and magnesium.

**3.3 Calibration curves and QC standards**

ACK calibration curves were prepared in the following concentrations: 3.91, 7.81, 15.6, 31.25, 62.5, 125, 250 and 500 µg/ml using cyclamate (200µg/ml) as internal standard (final concentrations). One standard curve along with at least three sets of quality control samples was prepared each day of analysis. The quality control concentrations were as follows: 31.25 g/ml, 125 g/ml and 500 g/ml for low quality control (QC1), middle quality control (QC2) and high quality control (QC3), respectively.
